# Drought and Warming‐Induced Drying Suppress Soil Respiration but Amplify Rewetting‐Induced Pulses in a Temperate Pasture

**DOI:** 10.1111/gcb.71005

**Published:** 2026-07-18

**Authors:** Pankaj Tiwari, Elise Pendall, Nicholas Wright‐Osment, Nor Azizah Kusai, Manjunatha H. Chandregowda, Awais Shakoor, Debjani Sihi, Sally A. Power, Eric A. Davidson, Catriona A. Macdonald

**Affiliations:** ^1^ Hawkesbury Institute for the Environment Western Sydney University Penrith New South Wales Australia; ^2^ Department of Environmental Systems Science ETH Zürich Zürich Switzerland; ^3^ Department of Plant and Microbial Biology and Crop and Soil Sciences, N.C. Plant Sciences Initiative North Carolina State University Raleigh North Carolina USA; ^4^ Appalachian Laboratory University of Maryland Center for Environmental Science Frostburg Maryland USA; ^5^ School of Agriculture and Environment University of Western Australia Perth Western Australia Australia

**Keywords:** biogeochemical hotspot, Birch effect, drying‐rewetting cycle, experimental warming, greenhouse gas emissions, pastures, rainfall alteration, soil carbon flux

## Abstract

As climate extremes intensify, interactions among environmental drivers are expected to alter soil respiration (SR) and its response to rewetting, increasing uncertainty in carbon‐climate feedbacks. However, the interactive effects of drought and warming, two commonly studied climate stressors, on SR remain elusive due to limited research and a lack of high‐resolution data. This study investigated overall SR (SR_overall_, encompassing both drying and rewetting phases) and rewetting‐induced respiration pulses (SR_pulse_), along with their apparent sensitivity to temperature and moisture under factorial combinations of rainfall and warming treatments in a field‐based climate‐manipulation experiment conducted in a temperate pasture system in southeastern Australia. Rainfall extremes were derived from 30 years of regional climate records, while warming was imposed as a continuous, year‐round increase of +3°C. An automated flux monitoring system was deployed to measure hourly SR across eight campaigns from October 2023 to November 2024. The drivers of SR_overall_ and SR_pulse_ were identified by analyzing climatic variables together with soil parameters from rhizosphere and non‐rhizosphere zones. Drought and warming consistently suppressed SR_overall_ but amplified SR_pulse_ and moisture sensitivity. The interactive effects of both treatments on SR_overall_ varied seasonally, shifting from additive in spring to antagonistic in summer and autumn, and synergistic in winter. Drought suppressed apparent temperature sensitivity (Q_10_), but warming effects on Q_10_ varied with moisture conditions. Soil temperature, moisture, and extractable C:N ratio from across rhizosphere and non‐rhizosphere soil were consistent predictors of SR_overall_ and SR_pulse_ but exerted opposing effects on the two components. These findings advance our understanding of how drought‐warming interactions shape both overall and pulse‐driven SR, providing a process‐based foundation for improving predictions of carbon‐climate feedbacks under intensifying climate extremes.

## Graphical abstract

A factorial rainfall × warming field experiment showed that drought and warming suppressed overall soil respiration but amplified rewetting‐induced CO_2_ pulses via contrasting effects of moisture, temperature, and substrate availability. Their interaction shifted seasonally, from additive (spring) to antagonistic (summer‐autumn) and synergistic (winter), highlighting complex carbon‐climate feedbacks.

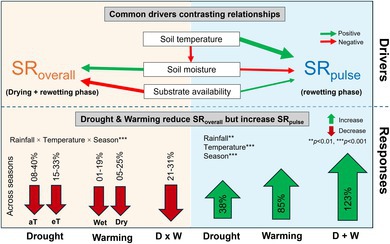

## 1 Introduction

Anticipated changes in climate means, particularly increased temperatures and altered rainfall patterns, are expected to intensify the frequency and severity of climate extremes, including heatwaves, heavy precipitation events, and prolonged droughts (Tripathy et al. 2023; AghaKouchak et al. 2020). These shifts may profoundly impact soil respiration (SR), a key component of the terrestrial carbon (C) cycle responsible for emissions of 107 Pg C annually (Liu et al. 2024). Consequently, understanding how SR responds to the combined effects of warming and predicted changes in precipitation is critical for projecting C‐climate feedbacks (Friedlingstein et al. 2025).

Warming generally stimulates SR, but this effect is partially offset by soil drying via warming‐induced evapotranspiration and intensified moisture deficits (Wang et al. 2014). When additional precipitation alleviates water limitation, warming and increased precipitation can enhance SR; however, only 14 factorial studies, involving both drivers, have been identified, highlighting the need for long‐term, multi‐factor investigations (Niu et al. 2008; Zhang, Li, et al. 2023). Meta‐analyses indicate strongly asymmetric outcomes depending on precipitation direction: warming with increased precipitation stimulates SR, whereas warming with reduced precipitation suppresses it, with CO_2_ losses exceeding those caused by drought alone (Yang et al. 2022). At the ecosystem scale, temperate forests and semi‐arid grasslands show antagonistic interactions in which drought constrains warming‐induced SR stimulation, producing combined effects that differ from those of either factor alone (Li et al. 2023; Schindlbacher et al. 2012). In nutrient‐rich managed montane grasslands, warming combined with elevated CO_2_ has been found to amplify drought effects on SR, producing synergistic negative effects that intensified both suppression and recovery dynamics (Meeran et al. 2021). Similarly, elevated CO_2_ and warming intensified drought‐induced suppression and post‐drought SR pulses, but by delaying drought onset, they eliminated any net reduction in annual SR under drought (Reinthaler et al. 2021), and in arid ecosystems, continued warming suppressed the drought‐induced reduction in SR (Yu et al. 2021). Together, these findings underscore the context‐dependency and uncertainty of SR responses to interacting climate drivers, highlighting the urgent need for long‐term, multi‐factor manipulation experiments across contrasting ecosystems and seasons.

Soil moisture availability often modulates SR responses to warming (Schindlbacher et al. 2012; Zhang, Zhou, et al. 2023). Under moist conditions, SR increases exponentially with temperature (Davidson and Janssens 2006; Tiwari, Bhattacharya, Rawat, Rai, and Talukdar 2021). In contrast, drought and warming‐induced soil drying suppress SR and its response to temperature by reducing substrate availability, plant productivity, and microbial activity (Widanagamage et al. 2025; Li et al. 2023, 2021; Bian et al. 2022; Morris et al. 2022). Additionally, temperature and moisture exert contrasting effects on the temperature sensitivity of SR; higher temperatures tend to reduce this sensitivity, while increasing soil moisture enhances it (Zhao et al. 2017; Peng et al. 2009; Tiwari, Bhattacharya, Rawat, and Talukdar 2021). However, how this sensitivity is affected by simultaneous changes in temperature and precipitation remains unclear.

Drying‐rewetting cycles introduce further complexity into SR dynamics. Rewetting dry soils often triggers sharp, pulsed increases in SR (SR_pulse_), a phenomenon known as the Birch effect, driven by microbial lysis, osmolyte release, and the sudden availability of previously inaccessible organic matter or labile C substrates (Dinh et al. 2018; Xiang et al. 2008; Birch 1958). These rewetting‐driven pulses represent “hot moments” in soil carbon cycling, characterized by short‐lived but disproportionately large biogeochemical fluxes (Anthony and Silver 2023; Fernandez‐Bou et al. 2020; Kuzyakov and Blagodatskaya 2015; Vargas et al. 2018). Recent work by Nguyen et al. (2025) demonstrates that rain‐induced SR accounts for nearly 17% of annual ecosystem respiration in global drylands, yet eddy‐covariance partitioning methods underestimate these pulses by 13%–18%, highlighting the need to better capture pulse‐driven losses in carbon budgets and models. The SR_pulse_ in dry soils can be 2–5 times higher than emissions from continuously moist soils (Sun et al. 2015), but their magnitude and persistence are modulated by rainfall‐temperature interactions and the ecosystem's moisture sensitivity. Although drought legacy effects on rewetting‐induced SR have been widely documented, how warming modifies these legacies, particularly in terms of SR_pulse_ magnitude, persistence, and post‐pulse recovery trajectories, remains poorly understood. Moreover, high‐frequency monitoring of SR during rewetting events is still rare, and conventional manual sampling approaches often fail to capture these short‐lived yet influential pulses (Li et al. 2023). Given the inherently episodic nature of these hot moments, high‐frequency (e.g., hourly or sub‐daily) automated measurements are essential for capturing transient responses and reducing uncertainties in the C budget estimates and model predictions.

Moreover, while the drivers of SR have been well studied, the specific drivers of SR_pulse_, especially those that integrate soil parameters from both rhizosphere and non‐rhizosphere zones, have received comparatively little attention. This represents a critical knowledge gap, as SR_pulse_ reflects rapid microbial and root responses to transient environmental shifts that are often masked when analyses focus solely on overall SR (SR_overall_), which represents the time‐averaged respiration including both steady and pulse periods. Importantly, SR may be governed by distinct biotic and abiotic factors in the rhizosphere and non‐rhizosphere soil, reflecting the differing ecological functions of these compartments. In the rhizosphere, SR is primarily influenced by root activity and substrate cycling, both tightly linked to microbial communities stimulated by root exudates (Jones et al. 2004). These root‐derived substrates enhance microbial respiration and enzyme production, making the rhizosphere a biogeochemical hotspot for rapid and dynamic C turnover. In contrast, SR in the non‐rhizosphere zone is more closely associated with the decomposition of soil organic matter and is regulated by broader physicochemical factors such as moisture, pH, and substrate availability (Lu et al. 2025; Allison and Vitousek 2005). This availability can be assessed in several ways, including total and extractable C, or by evaluating stoichiometric balances such as total C:N or extractable C:N ratios. The latter often provides a more integrative indicator than C alone, as it reflects both energy supply and nutrient balance, which together regulate microbial metabolism, enzyme production, and C use efficiency (Haney et al. 2012; Manzoni et al. 2012). Distinguishing these zone‐specific drivers is crucial for accurately predicting SR under fluctuating climate conditions. This spatial heterogeneity aligns with the concept of biogeochemical “hotspots,” where localized zones such as the rhizosphere exhibit disproportionately high rates of microbial activity and carbon turnover relative to surrounding bulk soil (Appuhn et al. 2004; Groffman et al. 2009; Kuzyakov and Blagodatskaya 2015; Vidal et al. 2018; Wen et al. 2022). Existing models commonly assume that the drivers of SR_overall_ also govern SR_pulse_ (Davidson et al. 2012; Manzoni et al. 2012). However, accumulating evidence suggests that SR_pulse_ can arise from distinct mechanisms, particularly where the rhizosphere acts as a temporary reservoir of labile carbon that is largely absent in non‐rhizosphere soil (Schimel 2018). Under such conditions, rewetting responses may be governed by different or even opposing stoichiometric constraints, such as shifts in extractable C:N ratio and microbial nutrient limitation, compared with those controlling SR_overall_. Identifying when drivers are shared versus decoupled between SR_overall_ and SR_pulse_ is essential for moving beyond empirical correlations toward mechanistic representations of multiple climate drivers in models. Together, this spatial–temporal coupling of biogeochemical hotspots (rhizosphere‐driven processes) and hot moments (rewetting‐induced pulses) provides a mechanistic framework for understanding how distinct controls regulate SR_overall_ versus SR_pulse_ under climate variability.

Pastures comprise around two‐thirds of the total agricultural land and have the potential to significantly contribute to and mitigate climate change (FAO 2023; Seó et al. 2017; Tubiello et al. 2007). These ecosystems store an estimated 120 Pg C as soil organic C, much of which is vulnerable to land‐use change, warming, and altered rainfall regimes (Lorenz and Lal 2018). For instance, over the period 1850–2015, global pastures contributed an estimated 16.3 Pg C in net emissions to the atmosphere (Houghton and Nassikas 2017). In Australia, where pastures dominate agricultural landscapes and climate variability is high, these systems are especially vulnerable to environmental change, highlighting their dual role as both potential C sinks and sources (Lee et al. 2013; Stokes et al. 2008). Yet, the feedback response of pasture SR, particularly rewetting‐driven pulses and their mechanistic controls, to the combination of drought and warming remains largely unexplored. Continuous, high‐resolution monitoring of SR, combined with climatic and soil assessments, can provide important insights into how SR responds to compounded climate stressors and help uncover the biogeochemical mechanisms underlying these responses (Bond‐Lamberty et al. 2024).

In this study, we conducted hourly SR measurements in a temperate pasture field experiment exposed to factorial climate manipulations: simulated rainfall extremes based on 30‐year historical data and a +3°C warming treatment. We assessed SR_overall_ and SR_pulse_, examined temperature and moisture sensitivity, and evaluated drivers using soil physico‐chemical parameters across rhizosphere and non‐rhizosphere zones. We addressed the following questions: (1) How do drought and warming, both independently and interactively, affect: (i) SR_overall_, (ii) the responses of SR to rewetting, particularly SR_pulse_ dynamics and the rate of SR decrease post‐pulse, and (iii) the temperature and moisture sensitivity of SR; (2) Do the environmental and stoichiometric drivers of SR_pulse_ diverge from those of SR_overall_, and how are these relationships influenced by the rhizosphere versus non‐rhizosphere soil properties?

## 2 Material and Methods

### 2.1 Study Site

This study was conducted at the PAstures and Climate Extremes (PACE) experimental field facility located at the Hawkesbury campus of Western Sydney University, Richmond, New South Wales, Australia (−33.60972, 150.73833, 25 m asl). The area receives a mean annual rainfall of 727 ± 36 mm, with substantial inter‐annual variability (Australian Government Bureau of Meteorology, Richmond‐UWS Hawkesbury Station, 1992–2021, Site number 067105). Rainfall is strongly seasonal, with summer (December–February) being the wettest period and winter (June–August) typically the driest. The mean annual temperature is 17.2°C (Bureau of Meteorology 2025). Seasonal daily mean maximum and minimum temperatures average 29.4°C/18.8°C in summer and 17.3°C/3.2°C in winter. Climate projections for the region indicate a potential increase in mean annual temperature of 2.5°C–4°C by the end of the 21st century (Sherwood et al. 2020; Pearce et al. 2007), with winter rainfall changes ranging from −20% to +5% under RCP_4.5_ and −40% to +5% under RCP_8.5_, and summer rainfall changes from −15% to +10% under RCP_4.5_ and −15% to +25% under RCP_8.5_ (Dey et al. 2019; Timbal 2004).

### 2.2 Experimental Design and Rainfall and Temperature Manipulation

The PACE experimental field facility consists of six replicate polytunnel shelters, each covered with a single layer of 250 μm polyethylene (Solarweave, GALE Pacific Pty Ltd., Australia). This study was conducted in only three of these shelters. Each shelter measures 48 m in length and 8 m in width, with a maximum height of 4.6 m. The shelters are oriented along a southwest to northeast axis, featuring open ends aligned with the prevailing wind direction to promote air circulation. The sides remain open to a height of 1.5 m along their entire length; however, experimental plots are set back from the shelter openings with buffer zones at the edges to prevent wind‐driven rainfall from entering the measurement area (Figure S1). Importantly, soil water content measurements at 5 cm depth did not show any variability attributable to natural rainfall, with changes occurring only following controlled irrigation events, confirming the effectiveness of the shelter design. In addition, each measurement plot was hydrologically isolated by a continuous root barrier installed to a depth of 90 cm, thereby preventing lateral water movement and cross‐treatment hydrological interference (Kite et al. 2025).

Climate manipulation studies were conducted at PACE from 2017 to mid‐2020 (Churchill et al. 2022). To minimize legacy effects from these previous experiments, the top 5 cm of soil was removed and replaced in February 2021 with a commercially available 80/20 sandy loam consisting of 80% double‐washed sand and 20% natural washed soil (Parklea Sand & Soil, Windsor, NSW, Australia). Following replacement, the soil was mechanically compacted to achieve a bulk density comparable to the surrounding field soil and to approximate natural field conditions. Soil chemical properties measured in August 2023 indicated low total carbon and nitrogen in the surface layer (0–5 cm: TC = 0.474 ± 0.038%, TN = 0.030 ± 0.002%, pH = 6.260 ± 0.085) relative to the 5–10 cm layer (TC = 0.652 ± 0.079%, TN = 0.043 ± 0.009%, pH = 5.047 ± 0.164).

Each shelter contained eight 4 × 4 m plots, four of which were divided into 2 × 2 m subplots sown with a legume‐grass mix of lucerne ( *Medicago sativa* ) and phalaris ( *Phalaris aquatica* ) in a 3:2 ratio in April 2022. These plots were harvested one or two times at the peak of their vegetative growth per season to a height of 5 cm aboveground using a sickle mower and hand shears. In Australian farming systems, pasture mixtures that include lucerne provide more stable forage production, improved water‐use efficiency, and greater resilience to drought and variable rainfall than lucerne monocultures, while also facilitating soil nitrogen availability for companion grasses (McCaskill et al. 2016).

The experiment employed a 2 × 2 factorial design to investigate SR responses to interacting climatic factors. Plots were randomly assigned to combinations of two simulated rainfall regimes: Wet and Dry, and two temperature levels: ambient and elevated temperature. This resulted in four treatment combinations: ambient temperature Wet (aTW), elevated temperature Wet (eTW), ambient temperature Dry (aTD), and elevated temperature Dry (eTD). Rainfall and temperature manipulation started in the first week of December 2022. To define experimental rainfall regimes, we analysed 30 years of rainfall data (1992–2021) from Richmond, NSW (Bureau of Meteorology station 067105; ~5 km from the site). From this record, the five wettest years (mean annual precipitation, MAP = 1060 mm) and the five driest years (MAP = 570 mm) were identified and used to determine seasonal rainfall amounts for the experimental treatments.

In order to generate representative irrigation schedules for both treatments, we selected two Dry (1994 and 2019) and two Wet (1999 and 2021) years from among the five driest/wettest years. Rainfall events in these years were categorized as < 2, 2–5, 5–10, 10–20 and > 20 mm, within each season (spring, summer, autumn, winter). We also summarized the total rainfall amount (in mm) in each size category and the average number of consecutive dry days between rainfall events. Our Wet and Dry rainfall schedules therefore reflect the seasonally explicit average number of rain events in each size class, and the mean interval between events, aligning with the past data from extreme Dry and Wet years. Relative treatment differences in rainfall amount in Dry and Wet treatment plots varied across seasons. During the study period, rainfall in the Dry treatment was 52%, 29%, 50%, and 33% lower than in the Wet treatment in spring, summer, autumn, and winter, respectively, resulting in an overall annual reduction of 42% (Figure S2, Table S1). We therefore define “drought” as a sustained seasonal reduction in rainfall amount and soil water content under the Dry treatment relative to the Wet treatment, rather than as a single extreme event. Rainfall was applied to each plot, predominantly at night, using a computer‐controlled spray irrigation system, as wind speeds are typically lower at night, which helps ensure uniform distribution of water within each plot.

A year‐round constant warming of +3°C above ambient plot surface temperature was achieved using infrared heaters (FTE 1000W, Ceramicx, Ireland) installed 1.4 m above ground. Target temperatures for these plots were controlled via feedback from IR sensors (SI‐100, Apogee Instruments, Logan, UT, USA) mounted at a height of 3.8 m, recording plot surface temperatures at 5‐min intervals. The heating treatment maintains daily temperature fluctuations in real‐time by warming plots in the elevated temperature treatment +3°C above recorded temperatures in ambient temperature plots.

### 2.3 Above Ground Biomass, SR, and Microclimate Measurements

In each subplot, one 11.4 cm tall PVC collar with a diameter of 20.1 cm was inserted at 2–3 cm depth in August 2023. Alongside plot‐level harvests, plants within these collars were clipped to < 2 cm before each flux measurement campaign to minimize CO_2_ flux from aboveground stems and leaves. Harvested plant material was dried at 70°C for 72 h then weighed to calculate the standing plant aboveground biomass (AGB). When multiple harvests occurred within a season, biomass was summed to obtain total AGB for that season.

Hourly SR was monitored, following 10 months of climate treatments, using twelve 8200‐104 Opaque Long‐Term Chambers linked to an LI‐7810 CH_4_/CO_2_/H_2_O Trace Gas Analyzer (LICOR Inc., Lincoln, NE, USA). These measurements were undertaken from 31 October 2023 to 28 November 2024 with a total of eight campaigns, each lasting 5–12 days. Measurements were campaign‐based rather than continuous. Within each campaign, SR was not measured if plant height inside the collars exceeded 5–8 cm. This minimized contributions from aboveground respiration and ensured that only SR was captured. Individual measurements lasted for 180 s, with a pre‐purge and post‐purge of 50 s each (purge refers to flushing the chamber with ambient air to remove residual gasses before and after measurement) and a dead band of 20 s (the initial period after chamber closure excluded from analysis to avoid artifacts caused by disturbance). Three replicate readings per treatment were collected within a 1‐h period. Post‐processing was conducted to ensure measurements were taken during a steady‐mixing state; dead band and stop time analyses were performed for each campaign separately in SoilFluxPro software (version 5.3.1, LICOR Inc.).

SR was calculated from the linear regression of CO_2_ concentration against time, as this approach consistently yielded higher *R* ^2^ values than exponential fits. Only SR measurements with *R* ^2^ > 0.9 were retained. The *R* ^2^ threshold was used solely as a quality‐control criterion and not for comparison of models or treatment effects. Additionally, negative CO_2_ flux values were excluded, based on the assumption that negligible photosynthesis occurred during chamber closure in the absence of light. Together, these criteria resulted in the removal of only two data points, retaining a total of 18,443 observations. Volumetric soil water content (SWC) and soil temperature (ST) at 5 cm depth were measured by a Stevens HydraProbe (Stevens Water Monitoring Systems Inc., Beaverton, OR, USA) connected to each long‐term chamber and a T107 temperature probe (Campbell Scientific, Logan, UT, USA), respectively.

### 2.4 Soil Sampling and Separation into Non‐Rhizosphere and Rhizosphere Soil

Soil sampling was conducted during March 2024, ~15 months after initiation of climate treatments. At each subplot, three soil cores were taken at 0–5 cm and 5–10 cm using a 5‐cm diameter stainless steel auger. The three cores were combined into one composite sample per depth and subplot. The samples were stored on ice and transported to the laboratory. Prior to sieving, soil was separated into rhizosphere and non‐rhizosphere subsamples. Briefly, roots with attached soil were picked and gently shaken to dislodge loose soil from the root surface. Soil that remained attached to the roots after shaking was classified as rhizosphere soil, whereas soil not adhering to the roots represented non‐rhizosphere soil (Phillips and Fahey 2006). Both subsamples were sieved through 2 mm, and visible fine roots were hand‐picked. Only fine roots (< 2 mm) were retained for further analyses, as they dominate root respiration (Zhang, Zhou, et al. 2023). Soil samples were stored at 4°C until further analyses.

### 2.5 Root Biomass and Soil Physico‐Chemical Parameters

Roots were washed and oven‐dried at 35°C for 5 days and weighed to determine dry root biomass. A low drying temperature was used to preserve samples for potential future isotopic analyses (Werth and Kuzyakov 2008). Five grams of soil was dried at 104°C for 24 h and weighed to calculate gravimetric water content. The soil was further ground using a ball mill MM400 (Retsch, Haan, Germany) at a frequency of 45 Hz for 45 s to measure total C and N using LECO TruMac CN Analyzer (Leco Corporation, St. Joseph, MI, USA), and the values were used to calculate the total C:N ratio. Microbial biomass C and N were assessed by chloroform fumigation extraction method (Joergensen and Mueller 1996). Two sets of glass vials containing 10 g of fresh soil were prepared. One set was fumigated with ethanol‐free chloroform in a desiccator for 45 h, while the other remained unfumigated. Both sets were extracted with 40 mL of 0.05 M K_2_SO_4_ (1:4 w:v) by shaking for 1 h and filtering through Whatman No. 42 filter paper. Extractable C and N in the extracts were analysed using a Shimadzu TOC‐L analyzer (Shimadzu Scientific Instruments Inc., Columbia, MD, USA). The unfumigated samples provided total extractable C and N (hereafter referred to as extractable C and extractable N) from which the extractable C:N ratio was calculated. Microbial biomass C and N were determined as the difference between fumigated and unfumigated extracts (Schmidt et al. 2021). Soil pH was measured in a 1:5 (w:v) fresh soil: 0.01 M CaCl_2_ suspension after shaking for 1 h, using a pH meter (S20 SevenEasy pH, Mettler Toledo, Greifensee, Switzerland) (Ahern et al. 1995). Microbial biomass C and N, and pH were assessed only for non‐rhizosphere soil due to insufficient rhizosphere soil quantity.

### 2.6 Microbial Extracellular Enzyme Activity

The activities of six enzymes involved in soil C, N, and P cycles including α‐1,4‐glucosidase (AG); β‐1,4‐glucosidase (BG); β‐D‐cellobiohydrolase (CB); β‐1,4‐xylosidase (XYL); β‐N‐acetylglucosaminidase (NAG); acid phosphatase (PH) were determined using a high‐throughput fluorometric microplate assay (Bell et al. 2013). Briefly, plates containing 800 μL soil slurry, 200 μL MUB, and 200 μL of MUB‐labeled substrates were sealed, incubated at 25°C for 3 h, and thereafter centrifuged for 3 min at ~2900×*g*. Supernatant, 250 μL, was read under a fluorometric plate reader (CLARIOstar Plus, BMG LABTECH, Ortenberg, Germany) at an excitation wavelength of 365 nm and an emission wavelength of 450 nm. Enzyme activity (nmol g^−1^ h^−1^) was averaged across two technical replicates prepared from the same soil slurry for subsequent analysis.

### 2.7 Data Analyses

All statistical analyses were performed using R 4.5.3 (R Core Team 2026). The rewetting response of SR under different treatments was assessed by calculating SR_pulse_, SR_peak_, and SR_decrease_. In this study, all rainfall events were treated as rewetting events, irrespective of preceding dry period length. SR_pulse_ (%) was assessed as the percentage increase in SR between 1 h before rewetting (time − 1) and SR_peak_ (μmol m^−2^ s^−1^), the SR upon rewetting (time 0). Antecedent SWC was used to account for legacy effects of prior moisture conditions rather than defining drought by a fixed duration. Since Wet and Dry treatments received different amounts of rainfall, and to compare the proportional response of SR to rewetting across treatments and campaigns, SR_pulse_ was normalized by rainfall quantity (termed as rain‐normalized SR_pulse_) for each specific pulse event. SR_decrease_ (μmol m^−2^ s^−1^ h^−1^) was calculated as the rate of post‐pulse decline in SR by dividing the difference between SR measured at the first and fifteenth hourly intervals by 15. A shorter window (< 12 h) often failed to capture the complete decay phase across campaigns, whereas a longer window (e.g., 24 h) increased the risk of interference from diurnal temperature fluctuations, weather changes, or additional rainfall events. Therefore, a 15‐h window was selected based on preliminary visualization of the dataset.

To assess the effects of rainfall, temperature, and season on SR, soil physico‐chemical properties, aboveground and belowground biomass and rewetting parameters, we employed linear mixed‐effects models using the lme4 and lmerTest packages (Bates et al. 2015; Kuznetsova et al. 2017). Experimental factors (rainfall, temperature, and season) and their interactions were included as fixed effects. Campaign and shelter were included as random intercepts to account for repeated measures and spatial heterogeneity when testing effects on SR_overall_, soil temperature, and moisture. For rewetting parameters, aboveground and belowground biomass, and soil parameters, only shelter was included as a random intercept as there were no repeated observations within campaigns. Models were fit using Restricted Maximum Likelihood (REML) with significance determined via Type III ANOVA and Satterthwaite's approximation for degrees of freedom (Kuznetsova et al. 2017; Pinheiro and Bates 2000). Normality of residuals was assessed using Q–Q plots, and data were log‐and‐sqrt‐transformed where necessary to meet test assumptions. Where interactions were significant, post hoc pairwise comparisons were conducted using Estimated Marginal Means (EMMs) (Lenth et al. 2025). These comparisons allowed for the decomposition of three‐way and two‐way interactions into meaningful treatment effects across seasons. Results were presented in terms of percentage drought and warming effects, which were calculated arithmetically from the raw data to represent observed magnitudes, while the associated statistical significance levels (*p*‐values) were derived from the corresponding mixed‐effects models (Li et al. 2023). Statistical significance was evaluated at *p* < 0.05, and all results presented are significant unless otherwise stated.

To formally test for non‐additivity, we calculated interaction contrasts (difference‐of‐differences); a significant interaction contrast (*p* < 0.05) indicated that the combined effect of warming and drought deviated significantly from the sum of their individual effects. We characterized the nature of these combined stressor effects (additive, synergistic, or antagonistic) by comparing the observed combined effect against an expected additive null model (Crain et al. 2008; Piggott et al. 2015). These combined effects were strictly based on effects relative to the no‐drought no‐warming treatment (aTW). The expected effect was calculated as the sum of the individual simple effects of warming (in the absence of drought) and drought (in the absence of warming) (Klein et al. 2004).

(1) $$\text{Combined effect expected}\;\left(\%\right)=\left(\left(\bar{\mathrm{aTD}}-\bar{\mathrm{aTW}}\right)+\left(\bar{\mathrm{eTW}}-\bar{\mathrm{aTW}}\right)\right)/\bar{\mathrm{aTW}}\times 100$$

(2) $$\text{Combined effect observed}\;\left(\%\right)=\left(\bar{\mathrm{eTD}}-\bar{\mathrm{aTW}}\right)/\bar{\mathrm{aTW}}\times 100$$

Relationships were classified based on the statistical significance of the interaction contrast from the LME model. Relationships where the interaction contrast was non‐significant (*p* > 0.05) were classified as additive. Significant interactions were classified as synergistic if the magnitude of the observed combined effect exceeded the additive expectation (|Observed| > |Expected|), or antagonistic if the combined effect was smaller in magnitude than expected (|Observed| < |Expected|).

Temperature‐SR and moisture‐SR relationships were assessed by performing exponential (Equation 3) and linear regression (Equation 4), respectively, across different treatments.

(3) $$\mathrm{SR}=\alpha e^{\mathrm{\beta t}}$$

(4) SR=a+bM

where *α*, *β, a*, and *b* are coefficients, and *t* and *M* represent soil temperature and moisture, respectively. Alternative functional forms (quadratic, sigmoidal, or broken stick) were also explored; however, they did not substantially improve the model fit (Figure S3). We retained the exponential and linear models as these have been widely used in previous studies to describe SR responses to temperature and moisture, respectively (Cook and Orchard 2008; Zhang et al. 2026). The apparent temperature sensitivity of SR (Q_10_) was calculated based on the coefficient *β* as:

(5) $$Q_{10}=e10^{\beta }$$

Moisture sensitivity was represented by the slope *b* of linear regression between SR and soil moisture (Equation 4). The temperature and moisture sensitivity of SR across the treatments were compared based on estimated marginal trends (slopes) obtained from the models using the emtrends function in the emmeans package (Lenth et al. 2025). To understand the role of moisture on the temperature‐SR relationship in each treatment, the flux data were first grouped into four moisture quartile ranges: low (< 25%), medium (25%–50%), high (50%–75%), and very high (> %75%), then exponential regression was conducted for each group and Q_10_ of SR was calculated as above.

To identify the drivers of SR_overall_ and SR_pulse_, theory‐informed path models were constructed and evaluated using structural equation model (SEM) analyses. The mean values of SR_overall_, SR_pulse_, microclimate variables, and edaphic properties (averaged across 0–5 cm and 5–10 cm soil depths) were calculated for each treatment and used in the model. Total potential extracellular enzyme activity was calculated as the sum of the activities of all measured enzymes and was subsequently included in the SEM analysis. Two separate SEM analyses were conducted: one incorporating rhizosphere and the other using non‐rhizosphere soil parameters. The comparative fit index (CFI), root mean square error of approximation (RMSEA), standardized root mean square residual (SRMR), and Akaike information criteria (AIC) values were compared for model selection (Kline 2016; Schermelleh‐Engel et al. 2003).

## 3 Results

### 3.1 Impacts of Drought and Warming on SR_overall_ , SWC, and ST

**FIGURE 1:**
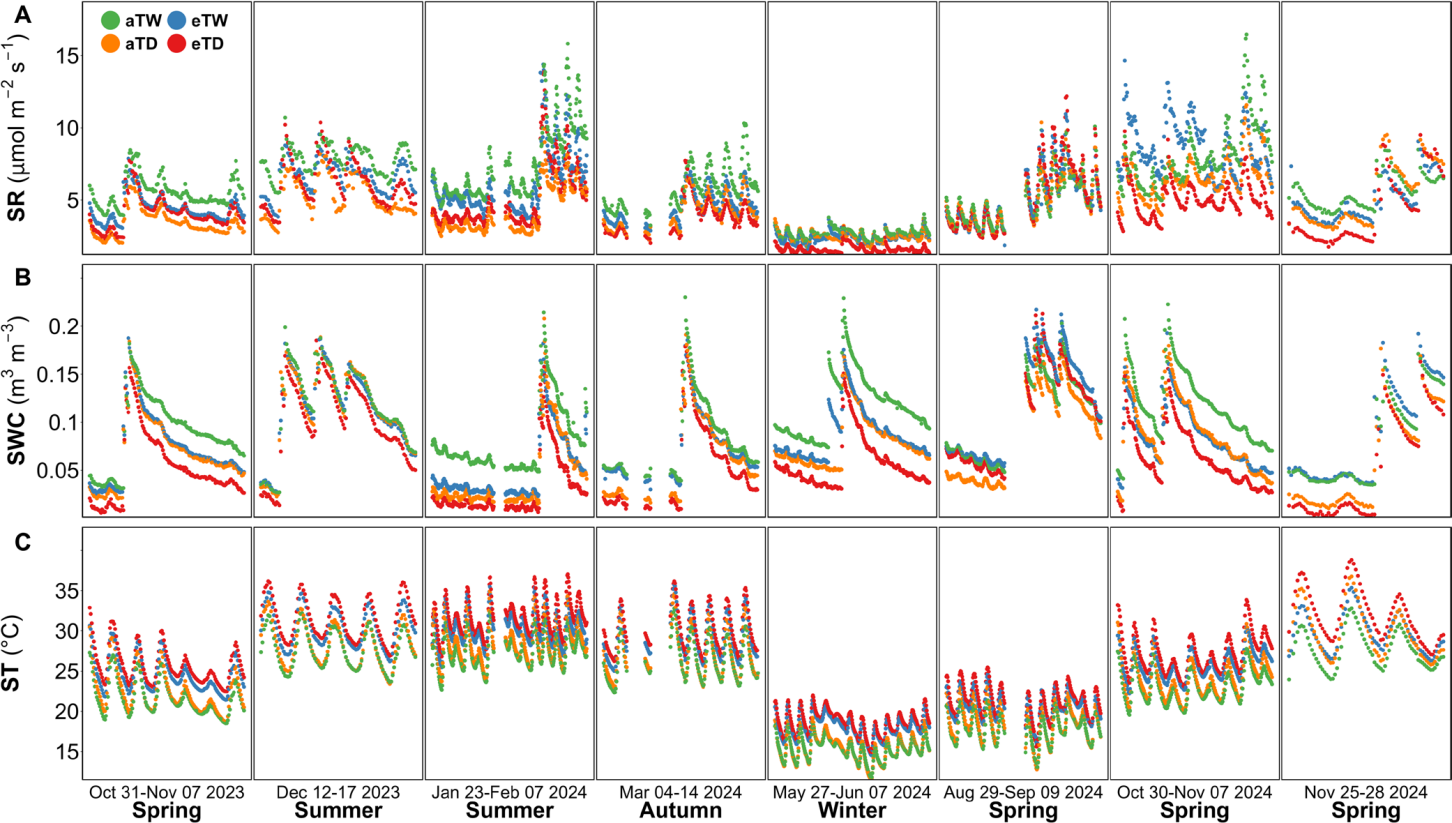
Soil respiration (SR) and microclimate under different treatments. Data are hourly means of (A) SR, (B) volumetric soil water content (SWC), and (C) soil temperature (ST). aTD, aTW, eTD, and eTW are treatments where aT and eT stand for ambient and elevated temperature, and D and W represent Dry and Wet, respectively.

SR_overall_, SWC, and ST exhibited distinct temporal patterns over the study period, with clear diurnal peaks in SR_overall_ and ST, and pronounced increases in both SWC and SR_overall_ following rainfall events (Figure 1). Throughout the study, mean SR_overall_ (μmol m^−2^ s^−1^) in aTW was highest, at 5.81 ± 0.04, followed by 5.25 ± 0.05 (eTW), 4.35 ± 0.03 (aTD), and 4.26 ± 0.04 (eTD). SR_overall_ was significantly influenced by a rainfall × temperature × season interaction (Table 1), reflecting strong seasonal variation in treatment responses. However, no rainfall × temperature interaction was detected in spring (Table 2). Drought consistently suppressed SR_overall_ across seasons by −8.1% to −40.4% under ambient and −14.5% to −23.1% under elevated temperature (Table 2 and Table S2). Under elevated temperature, drought‐induced reductions were attenuated in summer and autumn but intensified in winter. Warming also reduced SR_overall_ under both Wet (−0.8% to –18.9%) and Dry (−5.6% to −24.6%) treatments across seasons, except in summer, when it increased SR_overall_ by 19.1% under the Dry treatment. Consequently, the nature of interactions varied seasonally, with additive effects in spring (−17.5% reduction), antagonistic effects in summer and autumn when observed combined reductions (−29.0% and −36.0%) were less severe than additive expectations (−57.2% and −41.5%), and synergistic effects in winter, when the observed combined reduction (−30.7%) exceeded the additive expectation (−18.0%).

**TABLE 1:** Main and interactive effects of rainfall, temperature, and season on SR_overall_, soil microclimate, aboveground biomass and rewetting response parameters.

Effect	SR_overall_	SWC	ST	AGB	SR_pulse_	Norm. SR_pulse_	SR_peak_	SR_decrease_
Rainfall	1892.2***	1898.7***	61.6***	7.9**	8.4**	42.0***	1.5 ns	0.2 ns
Temperature	579.4***	674.8***	1333.3***	4.3*	23.7***	27.3***	3.4 ns	7.5**
Season	170.0***	446.4***	32.3***	7.3***	8.6***	3.2*	29.7***	5.6**
Rainfall × temperature	4.3*	2.6 ns	9.1**	0.8 ns	0.0 ns	3.6 ns	0.3 ns	0.0 ns
Rainfall × season	55.7***	14.5***	1.0 ns	1.2 ns	1.3 ns	3.4*	0.5 ns	0.3 ns
Temperature × season	63.3***	22.4***	0.3 ns	0.2 ns	0.1 ns	0.3 ns	0.3 ns	0.4 ns
Rainfall × temperature × season	106.4***	6.6***	1.3 ns	0.8 ns	0.9 ns	0.9 ns	0.1 ns	0.1 ns

*Note:* Values presented are *F*‐statistics and associated *p*‐values derived from linear mixed‐effects models. Significance levels: *p < 0.05, **p < 0.01, ***p < 0.001; ns, not significant.

Abbreviations: AGB, aboveground biomass; Norm. SR_pulse_, rain‐normalized SR_pulse_; SR, soil respiration; SR_decrease_, SR decrease rate post pulse; SR_peak_, peak SR upon rewetting; SR_pulse_, SR increase upon rewetting; ST, soil temperature; SWC, soil water content.

**TABLE 2:** Drought and warming effects on SR_overall_, soil microclimate, aboveground biomass, and rewetting response parameters.

Factor	Season	Drought effect		Warming effect		Combined effect		Interaction	
		aT	eT	Wet	Dry	Expected	Observed	Sig.	Type
SR_overall_	Spring	−16.7%***	−20.7%***	−0.8%***	−5.6%***	−17.5%	−21.3%	ns	Additive
	Summer	−40.4%***	−14.5%***	−16.9%***	19.1%***	−57.2%	−29.0%	***	Antagonistic
	Autumn	−22.6%***	−21.0%***	−18.9%***	−17.3%***	−41.5%	−36.0%	*	Antagonistic
	Winter	−8.1%***	−23.1%***	−9.9%***	−24.6%***	−18.0%	−30.7%	***	Synergistic
SWC	Spring	−20.3%***	−21.3%***	−8.7%***	−9.9%***	−29.0%	−28.2%	ns	Additive
	Summer	−28.0%***	−28.2%***	−25.2%***	−25.3%***	−53.2%	−46.2%	**	Antagonistic
	Autumn	−25.2%***	−30.0%***	−15.4%***	−20.8%***	−40.6%	−40.8%	ns	Additive
	Winter	−33.8%***	−29.9%***	−22.9%***	−18.3%***	−56.7%	−45.9%	**	Antagonistic
ST	Overall	0.4°C***	0.8°C***	2.6°C***	3.0°C***	3.0°C	3.4°C	**	Synergistic
AGB	Overall	−45.7%**		−31.7%*		−77.4%		ns	Additive
SR_pulse_	Overall	37.8%**		84.7%***		122.5%		ns	Additive
Norm. SR_pulse_	Spring	204.2%***		85.4%***		289.6%		ns	Additive
	Summer	73.6%**				159.0%		ns	Additive
	Autumn	85.8% ns				171.2%		ns	Additive
	Winter	192.7%**				278.0%		ns	Additive
SR_peak_	Overall	−15.9% ns		26.0% ns		10.0%		ns	Additive
SR_decrease_	Overall	−14.0% ns		101.9%**		87.8%		ns	Additive

*Note:* Effect sizes are expressed as percentage changes (except for ST), calculated directly from raw treatment means to represent observed magnitudes. Statistical significance (*p*‐values) was determined using post hoc pairwise comparisons based on estimated marginal means provided in Table S1. Significance levels: *p < 0.05, **p < 0.01, ***p < 0.001; ns, not significant. Pairwise comparisons were conducted only for parameters where significant interactions were detected in Table 1. aT and eT stand for ambient and elevated temperature, and D and W represent Dry and Wet, respectively.

Abbreviations: AGB, aboveground biomass; Norm. SR_pulse_, rain‐normalized SR_pulse_; SR, soil respiration; SR_decrease_, SR decrease rate post pulse; SR_peak_, peak SR upon rewetting; SR_pulse_, SR increase upon rewetting; ST, soil temperature; SWC, soil water content.

SWC showed similar sensitivity to treatments, with drought consistently reducing soil moisture by −20.3% to −33.8% under ambient and −21.3% to −30.0% under elevated temperature (Tables 1 and 2). Warming also reduced SWC under both Wet and Dry treatment, although to a lesser extent (−8.7% to −25.2%). Interaction types were additive in spring and autumn, but antagonistic in summer and winter, as the observed combined reductions (−46.2% and −45.9%, respectively) were smaller than expected (−53.2% and −56.7%), indicating that warming partially offset drought‐induced declines in soil moisture during these seasons.

There was a significant rainfall × temperature interaction on ST, with warming increasing ST under both Wet (2.6°C) and Dry (3.0°C) treatments (Tables 1 and 2). While drought alone increased temperatures modestly (0.4°C–0.8°C), the combined treatment triggered a synergistic 3.4°C rise, above the 3.0°C predicted from additive effects.

### 3.2 Impacts of Drought and Warming on Aboveground and Belowground Biomass, Soil Physico‐Chemical Properties and Microbial Extracellular Enzyme Activity

Rainfall, temperature, and season exerted independent effects on aboveground biomass, with no significant interactions observed between the factors (Figure S4A; Table 2). Drought and warming significantly reduced aboveground biomass by −45.7% and −31.7%, respectively (Table 2). Furthermore, the combination of these factors led to an additive reduction of −77.4%. No significant effects of drought and warming were observed on belowground biomass, although this tended to be higher under Dry treatments at the 5–10 cm soil depth (*p* = 0.054), particularly under the combined drought and warming treatment (eTD) (Figure S4B). Warming significantly increased soil total N in the 5–10 cm layer and decreased extractable C in the 0–5 cm layer (Figure S5). No other significant treatment effects were detected on soil physicochemical properties including total C:N and extractable C:N ratios. Rainfall significantly influenced the activity of three microbial extracellular enzymes, BG, CB, and XYL, with drought leading to greater enzyme accumulation in the rhizosphere (Figure S6).

### 3.3 Impacts of Drought and Warming on SR Response to Rewetting

**FIGURE 2:**
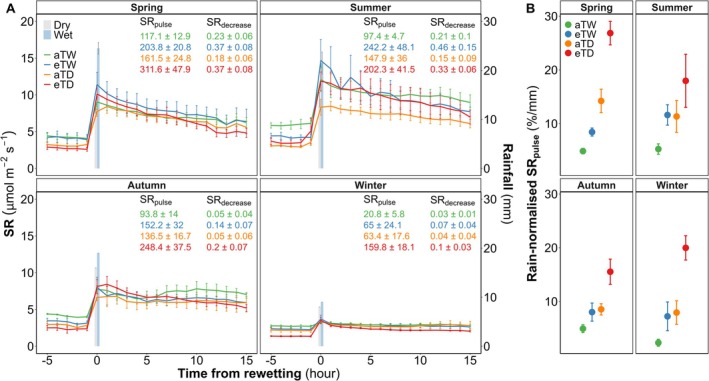
Soil respiration (SR) response to rewetting. (A) Average SR across campaigns and soil collars during a rewetting event, for each season; bars show standard errors. Insets indicate values of SR_pulse_ (%), the increase in SR within 1 h of rewetting, and SR_decrease_ (μmol m^−2^ s^−1^ h^−1^) is the rate of decrease in SR after rewetting‐induced peak. Vertical bars depict quantity of rainfall in Wet (blue) and Dry (gray) treatments. (B) Rain‐normalized SR_pulse_. Normalization was achieved by dividing each SR_pulse_ by the rainfall amount received by the treatment during that event. Colors depict the respective treatment aTD, aTW, eTD, and eTW, where, aT and eT stands for ambient and elevated temperature, and D and W represent Dry and Wet, respectively.

Automated high‐frequency SR measurements aided in capturing the SR response to rewetting in the field. SR_pulse_ was consistently smallest in ambient temperature Wet (aTW) plots and largest under the combination of elevated temperature and Dry (eTD) treatments. The magnitude of this SR_pulse_ followed a distinct seasonal gradient: 117.1%–311.6% in spring (aTW and eTD), 97.4%–242.2% in summer, 93.8%–248.4% in autumn, and 20.8%–159.8% in winter (Figure 2A), reflecting increases in SWC (SWC_pulse_) upon rewetting (Figure S8). Rainfall, temperature, and season each had independent effects on SR_pulse_, with no significant interactions among them. Both drought and warming increased SR_pulse_, although the effect of warming (+84.7%) was larger than that of drought (+37.8%) (Tables 1 and 2). When combined, the two factors acted additively, increasing SR_pulse_ by 122.5%.

When expressed per unit rainfall, SR_pulse_ was influenced by a significant interaction between rainfall and season, while temperature exerted only a main effect (Figure 2B and Table 1). Drought increased rain‐normalized SR_pulse_ by 204.2%, 73.6%, and 192.7% during spring, summer, and winter, respectively, with no significant effect in autumn (Table 2). In contrast, warming increased rain‐normalized SR_pulse_ by 85.4% irrespective of season. The combined effect of drought and warming resulted in increases ranging from 159% to 289.6%. Moreover, SR_peak_ did not show any treatment effects indicating similar SR under all treatment groups upon rewetting. Following the peak, SR declined within 15 h by −31% to −51% in spring, −20% to −42% in summer, −8% to −34% in autumn, and by −14% to 40% in winter. SR_decrease_ was affected by independent effects of temperature and season with no significant effects of rainfall or interactions (Table 1). Warming increased the rate of decrease in SR (SR_decrease_) post rewetting by 101.9% irrespective of rainfall treatment (Table 2).

### 3.4 Impacts of Drought and Warming on Temperature and Moisture Sensitivity

**FIGURE 3:**
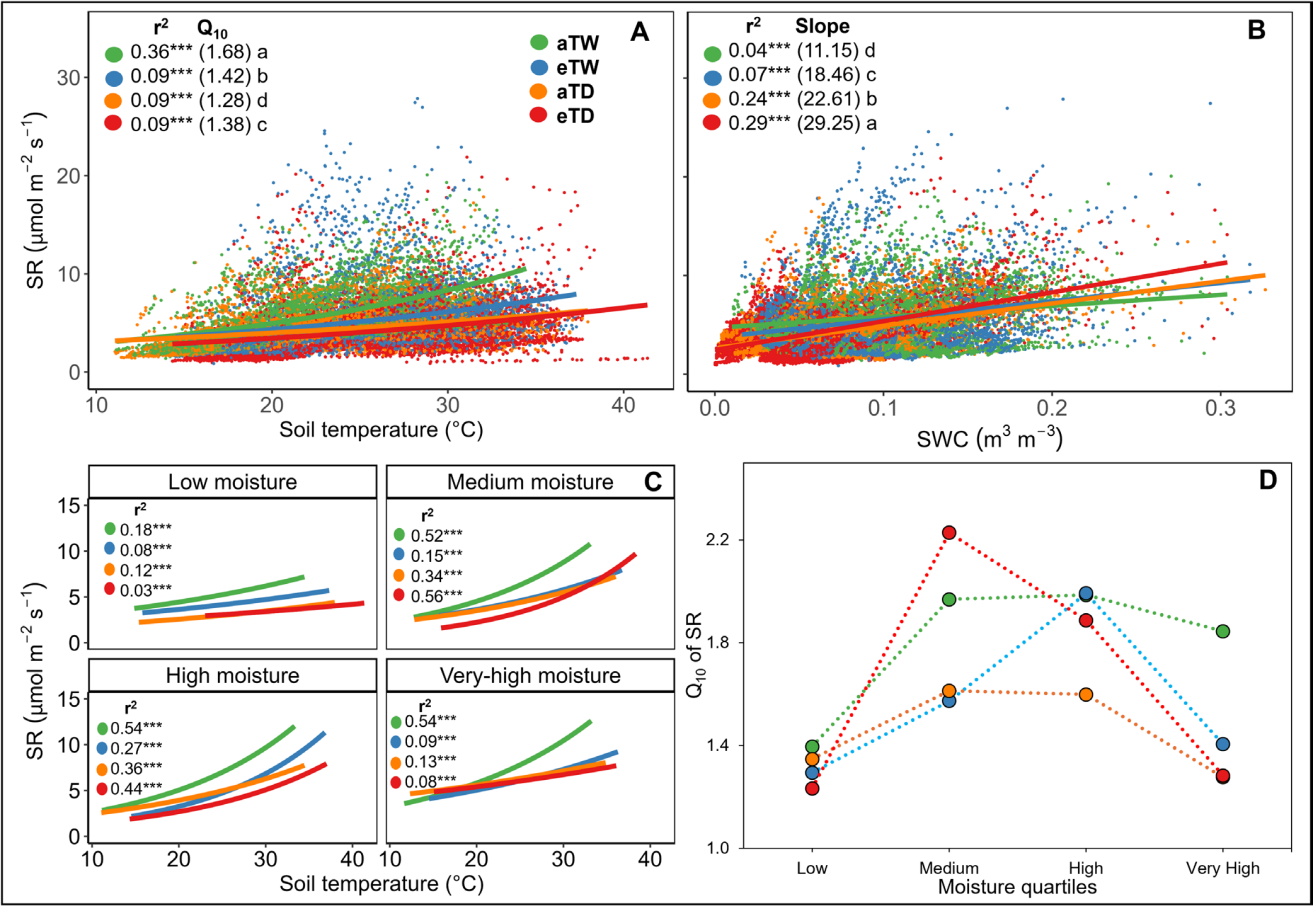
Effects of drought and warming on temperature and moisture relationships with soil respiration (SR). (A) Exponential temperature‐SR, and (B) linear moisture‐SR, relationships across different treatments as per Equations (3) and (4), respectively. Temperature sensitivity (Q_10_) and moisture sensitivity are shown in brackets. Treatment differences were assessed using estimated marginal trends (slopes) derived from linear models with the *emtrends* function. Different lower‐case letters indicate significant differences among treatments (*p* < 0.05). (C) Exponential temperature‐SR relationship across four soil moisture quartiles (low, medium, high, and very high) under each treatment. (D) Q_10_ of SR across soil moisture quartiles for each treatment (*n* = 3). *** represents significance of the regression models at *p* < 0.001. aTD, aTW, eTD, and eTW are treatments where aT and eT stands for ambient and elevated temperature, and D and W represent Dry and Wet, respectively.

Relationships of SR with temperature and moisture were statistically significant but relatively weak (Figure 3A,B). Under aTD, eTD, and eTW, soil temperature explained only 9% of the variability in SR, compared with 36% under aTW. (Figure 3A). The apparent temperature sensitivity of SR (Q_10_) ranged from 1.27 to 1.68 and differed significantly among treatments. Drought reduced Q_10_ under both ambient (23.7%) and elevated temperature (2.8%), although the magnitude of reduction was much smaller under the latter. In contrast, warming reduced Q_10_ under the Wet treatment by 15.7% but increased it under the Dry treatment by 7.5%.

SWC explained 4%, 7%, 24%, and 29% of the variation in SR under aTW, eTW, aTD, and eTD treatments, respectively (Figure 3B). The moisture sensitivity of SR increased progressively from aTW < eTW < aTD < eTD, with the driest treatment (eTD) exhibiting 260% greater moisture sensitivity than the wettest treatment (aTW). Drought increased moisture sensitivity under both ambient (102.8%) and elevated temperature (58.5%), while warming also enhanced moisture sensitivity by 65.5% under Wet and 29.4% under Dry treatments. To assess how soil moisture influences the apparent temperature sensitivity of SR, the data were divided into four SWC quartile ranges: low, medium, high, and very high. The relationships between SR and soil temperature were then examined within each range across treatments (Figure 3C). The apparent temperature sensitivity varied substantially with soil moisture. The SR‐ST relationship was strongest in the medium and high SWC quartiles for all treatments, with the highest Q_10_ observed under medium moisture for each treatment: 2.01 (aTW), 1.68 (eTW), 1.71 (aTD), and 2.22 (eTD) (Figure 3D). In contrast, Q_10_ values were consistently lowest under very high and low SWC quartiles.

### 3.5 Drivers of SR_overall_ and SR_pulse_ From Rhizosphere and Non‐Rhizosphere Soil

**FIGURE 4:**
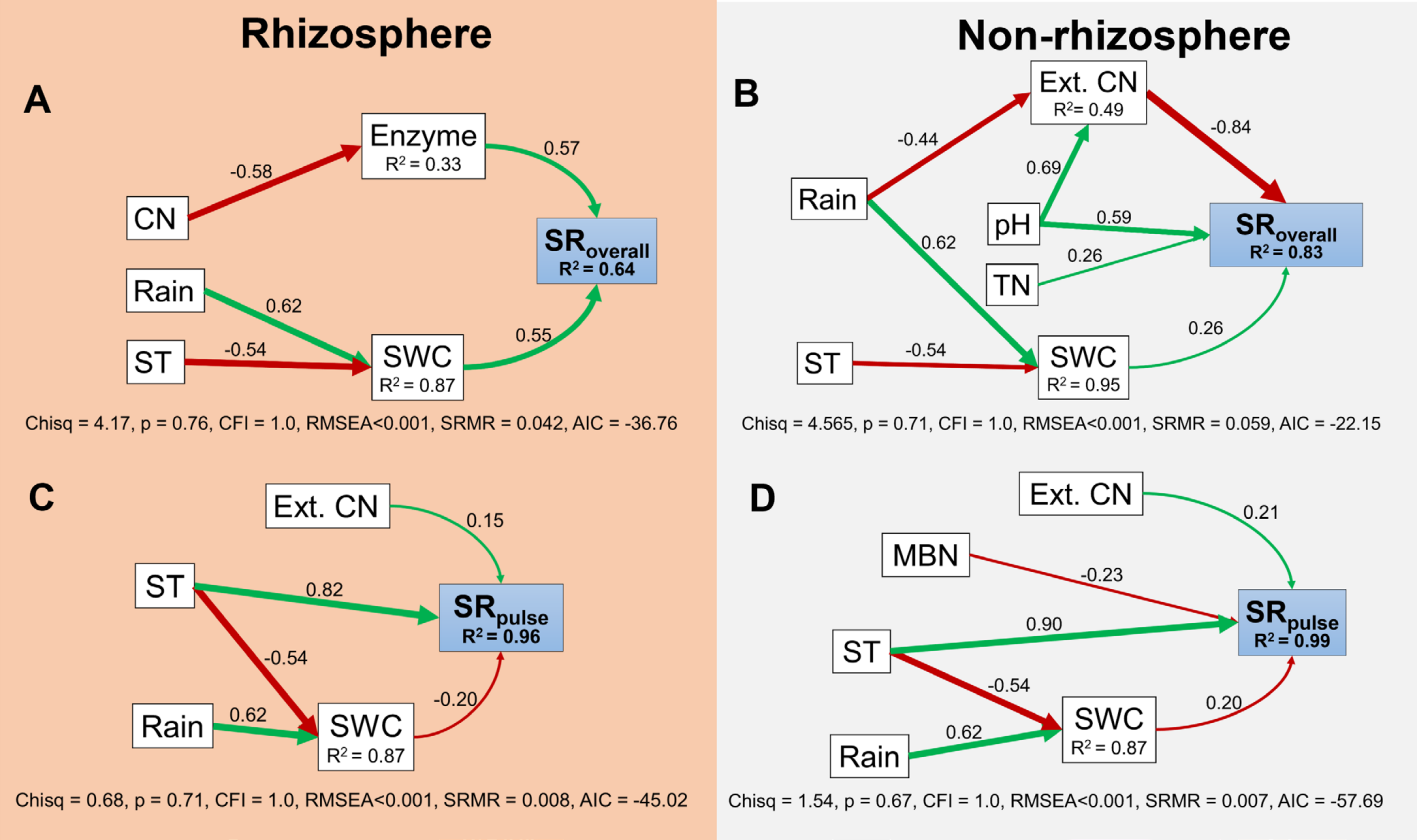
Direct and indirect factors driving SR_overall_ and SR_pulse_. Structural equation model (SEM) analyses of effects of selected climatic and edaphic parameters (from rhizosphere and non‐rhizosphere zones) on SR_overall_ (A, B), and SR_pulse_ (C, D). SR_overall_ represents mean SR across the study and includes both drying and rewetting periods, while SR_pulse_ only includes mean SR increase (%) upon rewetting. Red and green arrows represent negative and positive relationships, respectively. The width of arrows is proportional to the strength of standardized path coefficients denoted by the numbers adjacent to the arrows. *R* ^2^ denotes the proportion of variance explained by the fixed factors. The comparative fit index (CFI), root mean square error of approximation (RMSEA), standardized root mean square residual (SRMR), and Akaike information criteria (AIC) value, of each model are displayed as performance parameters.

Aboveground biomass was significantly related to SR_overall_ in all treatment groups except eTW (Figure S7). However, it was excluded from the SEM analysis due to collinearity and its lower explanatory power compared with other parameters. When considering effects of rhizosphere properties, SR_overall_ was directly and positively influenced by enzyme activity and SWC (Figure 4A). ST and total C:N ratio had indirect negative effects, acting through SWC and enzyme activity, respectively. Additionally, rainfall positively influenced SR_overall_ via its effect on SWC. Considering effects of non‐rhizosphere soil properties, SR_overall_ was primarily driven by direct negative effects of extractable C:N ratio and positive effects of pH, with SWC and total N also contributing positively (Figure 4B). ST and rainfall influenced SR indirectly via changes in SWC, as was found for the rhizosphere. These models explained 64% and 84% of the variance in SR_overall_, respectively.

Importantly, the drivers that influenced SR_overall_ generally affected SR_pulse_ in the opposite direction and were largely consistent between rhizosphere and non‐rhizosphere soils, except for microbial biomass N in non‐rhizosphere, which showed a negative relationship with SR (Figure 4C,D). ST had a strong positive influence on SR_pulse_, both directly and indirectly through its negative relationship with SWC. SWC, in turn, had a negative effect on SR_pulse_; thus, while rainfall increased SWC, it indirectly suppressed SR_pulse_. Extractable C:N ratio also positively influenced SR_pulse_. These models accounted for 96%–99% of the variation in SR_pulse_.

**FIGURE 5:**
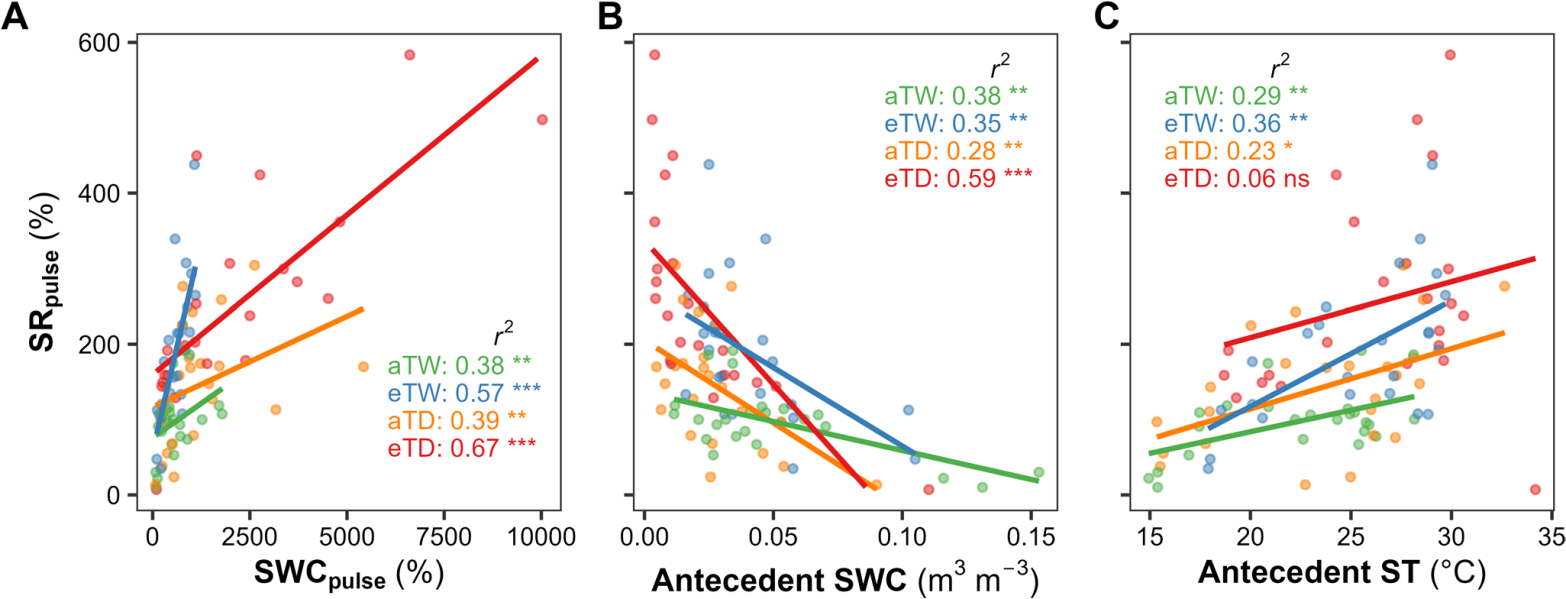
Influence of pulse and antecedent moisture and antecedent temperature on SR_pulse_ across different treatments. Linear relationship between SR_pulse_ and (A) soil water content pulse (SWC_pulse_), (B) antecedent SWC, and (C) antecedent soil temperature (ST). ***p < 0.001, **p < 0.01, *p < 0.05 and ns indicates non‐significant. SWC_pulse_ represents increase in SWC (%) upon rewetting. Antecedent denotes conditions 1 h prior to rewetting event. aTD, aTW, eTD, and eTW are treatments where, aT and eT stands for ambient and elevated temperature, and D and W represent Dry and Wet, respectively.

The influences of SWC_pulse_ (increase in SWC upon rewetting), antecedent soil moisture and antecedent temperature on SR_pulse_ were also evaluated across treatments to further examine the role of moisture pulses and legacy soil climate conditions (Figure 5). These variables were not included in the SEM analyses because their effects on SR were weaker than those of mean soil temperature and moisture. Nevertheless, SR_pulse_ showed strong relationships with soil moisture dynamics, increasing with SWC_pulse_, decreasing with antecedent SWC, and responding positively to antecedent temperature in all treatments except eTD.

## 4 Discussion

In our study, drought and warming suppressed SR_overall_, with the nature of their interactions varying seasonally. Conversely, drought and warming amplified SR_pulse_. These divergent responses were accompanied by shifts in sensitivities: drought reduced apparent temperature sensitivity (Q_10_), warming effects on Q_10_ depended on moisture conditions, and respiration became more responsive to changes in soil water availability under both treatments. Temperature, soil moisture, and extractable C:N ratio consistently regulated both SR_overall_ and SR_pulse_ across rhizosphere and non‐rhizosphere zones, but in opposing directions, highlighting a fundamental decoupling between baseline and pulse‐driven soil carbon fluxes under climate extremes.

### 4.1 Drought and Warming Suppress SR_overall_

Drought and warming consistently suppressed SR_overall_ across most seasons, primarily through coupled limitations of soil moisture and substrate availability. Low soil moisture disrupts the aqueous connectivity of soil pore networks, impeding solute diffusion and constraining microbial access to dissolved organic carbon, a mechanism well described by percolation theory and empirically supported across ecosystems (Davidson and Janssens 2006; Moyano et al. 2013; Schimel 2018; Tecon and Or 2017). Warming amplified these effects likely by accelerating evapotranspiration, deepening soil moisture deficits, and compounding hydraulic limitation (Denissen et al. 2024). Substrate limitation further contributed to the suppression of SR_overall_, as indicated by the significant positive relationship between aboveground biomass (AGB) and SR_overall_ in our study. Both drought and warming independently reduced AGB, likely reducing litter inputs and root‐derived carbon supply to the soil, while warming also reduced extractable C:N, collectively constraining the substrate pools available to sustain microbial and autotrophic respiration (Chandregowda et al. 2023; Suseela et al. 2012; Walker et al. 2018). These constraints also dampened the Q_10_ of SR. As soils dry, restricted enzyme‐substrate encounters limit the expression of intrinsic enzymatic thermodynamics in observed CO_2_ fluxes. Consistent with the Dual Arrhenius–Michaelis–Menten (DAMM) framework, substrate limitation imposes a kinetic ceiling on respiration, suppressing apparent Q_10_ even when enzyme‐level temperature sensitivity remains intact (Davidson et al. 2012; Davidson and Janssens 2006; Pallandt et al. 2022; Sierra et al. 2015; Sihi et al. 2018). Although extracellular enzyme activity increased under drought, this likely reflects microbial carbon foraging rather than enhanced metabolic throughput, as CO_2_ production remained constrained by water availability (Allison and Vitousek 2004; Schimel 2018). Similar reductions in Q_10_ during dry years have been reported in long‐term forest studies, underscoring moisture‐mediated suppression of temperature sensitivity as a general feature of ecosystem carbon cycling (Kurganova et al. 2022).

### 4.2 Drought and Warming Amplify SR_pulse_

Despite suppressing SR_overall_, both drought and warming increased the moisture sensitivity of SR and amplified SR_pulse_ across seasons, highlighting contrasting responses of overall and pulse‐driven respiration to climate extremes. This divergence suggests that interacting climate drivers may suppress baseline respiration while enhancing the relative contribution of rewetting‐driven hot moments to short‐term soil carbon fluxes (Hoover et al. 2016; Manzoni et al. 2020; Metz et al. 2023; Schindlbacher et al. 2012). This pattern is consistent with the classical Birch effect (Birch 1958), whereby drying promotes the accumulation of labile substrates and microbial osmolytes that are rapidly mineralized upon rewetting (Unger et al. 2010; Warren and Manzoni 2023; Xiang et al. 2008). In our experiment, lower antecedent SWC and larger increases in soil moisture following rainfall (SWC_pulse_) corresponded with stronger SR_pulse_, indicating that pulse magnitude is strongly regulated by both prior soil moisture and the extent of rewetting (Barnard et al. 2020; Jarvis et al. 2007; Lado‐Monserrat et al. 2014; Liu et al. 2022; Manzoni et al. 2020). Mechanistically, these pulses are consistent with osmotic stress in drought‐affected microbial communities, which drives the accumulation of intracellular osmolytes (e.g., trehalose, ectoine) and other compatible solutes that are rapidly consumed as labile substrates upon rewetting, together with extracellular carbon mobilized by the physical effects of wetting (Bouskill et al. 2016, 2025; Slessarev and Schimel 2020; Warren and Manzoni 2023). In support of this interpretation, studies have shown that both microbial and root respiration increase following rewetting, but microbial respiration accounts for most of the CO_2_ pulse, highlighting the dominant role of microbial processes in driving SR_pulse_ (Waring and Powers 2016; Yu et al. 2021). Additionally, aggregate disruption and capillary refilling can mobilize previously protected organic matter, further stimulating respiration pulses (Najera et al. 2020; Navarro‐García et al. 2012).

Warming amplified SR_pulse_ beyond the effects of drought alone, likely because warming‐induced soil drying increased microbial sensitivity to rewetting and, as shown in warming experiments, enhanced microbial respiration and substrate turnover under higher temperatures (Liang et al. 2023; Yang et al. 2023; Yu et al. 2021). Warming has also been shown to enhance SR responses following rewetting by increasing the resilience of respiration after drought and strengthening the temperature dependence of post‐rewetting CO_2_ emissions (Li et al. 2025; Yu et al. 2021). Substrate availability also acted as an important modulator of pulse magnitude. In our nutrient‐poor, low‐organic‐matter system, rewetting pulses represented critical windows for microbial activity and carbon release, consistent with work showing that CO_2_ pulses after dry periods are tightly controlled by labile substrate pools that accumulate under dry conditions (Barnard et al. 2020; Dong et al. 2021; Rosinger et al. 2023; Waring and Powers 2016). The observed increase in Q_10_ from low to moderate soil moisture suggests that rewetting alleviated diffusion constraints on substrates and microbial movement, thereby restoring effective reaction–diffusion coupling and enhancing the apparent temperature responsiveness of microbial respiration (He et al. 2024; Liu et al. 2016; Zhou et al. 2014). However, the rapid post‐pulse decline (SR_decrease_) under warming indicates faster exhaustion of small, labile C pools in a system already constrained by low biomass and extractable carbon, consistent with substrate depletion theory and warming studies in substrate‐limited soils (Du et al. 2025; He et al. 2023; Walker et al. 2018).

### 4.3 Nature of Drought and Warming Interactions Differ Across Seasons

The effect of drought and warming on SR_overall_ shifted from additive in spring to antagonistic in summer and autumn, implying that drought‐induced reductions in SR_overrall_ were attenuated under elevated temperature. This pattern likely reflects two mechanisms. First, soil water content reached very low levels (~0.05 m^3^ m^−3^) during these seasons, limiting further moisture reduction under combined treatments and resulting in additive or less‐than‐additive declines in SWC. Second, higher soil temperatures (25.7°C–31.6°C) enhanced SR_pulse_ following rewetting as Q_10_ improved under higher moisture, particularly under combined drought and warming. Because both drivers increased pulse magnitude, these rewetting‐induced respiration bursts partially compensated for drought‐driven reductions in SR_overall_, thereby weakening the expected additive suppression as suggested by studies (Liang et al. 2024; Reinthaler et al. 2021; Schindlbacher et al. 2012; Yu et al. 2021). This pulse‐driven compensation ultimately produced an overall antagonistic response.

By contrast, there was a clear synergistic suppression of SR_overall_ in winter, where drought‐induced reductions were intensified under elevated temperature. This was largely due to limited rewetting events in winter (Table S1), which reduced the occurrence and intensity of SR_pulse_, and low temperatures that constrained microbial reactivation, diminishing SR_pulse_'s contribution to SR_overall_ (Guan et al. 2023; Yuste et al. 2003). Such synergistic suppression is consistent with observations from cold or seasonally dry ecosystems, where warming intensified moisture limitation and disproportionately reduced SR under combined stress (Li et al. 2023). Collectively, these patterns demonstrate that seasonal context, specifically the interplay among rainfall frequency, temperature, and carbon supply, determines whether rainfall × temperature interactions emerge as additive, antagonistic, or synergistic, rather than the mere co‐occurrence of climatic drivers.

### 4.4 Common Drivers Exert Contrasting Effects on SR_overall_ and SR_pulse_

Contrasting responses of SR_overall_ and SR_pulse_ revealed a fundamental duality in soil carbon efflux regulation. Soil moisture associated positively with SR_overall_ indicating maintained water‐film continuity around soil particles, thereby facilitating substrate diffusion and microbial metabolic activity (Moyano et al. 2013; Schimel 2018; Stark and Firestone 1995). In contrast, it inversely regulated SR_pulse_ likely by governing the accumulation of labile substrates during antecedent drying: prolonged moisture deficits permit greater buildup of mineralizable carbon, resulting in disproportionately large pulses upon rewetting (Patel et al. 2021). Temperature similarly exerted opposing controls. It indirectly suppressed SR_overall_ probably through warming‐induced soil desiccation (Reichstein et al. 2013), yet enhanced SR_pulse_ likely by accelerating microbial metabolism once moisture constraints are relieved, with carbon turnover rates increasing sharply as soils transition from dry to moist conditions (Borken and Matzner 2009; Li et al. 2025; Manzoni et al. 2020; Yu et al. 2021). High extractable C:N ratio constrained SR_overall_ under chronic stress, consistent with stoichiometric nitrogen limitation reducing microbial carbon‐use efficiency and mineralization rates (Cleveland and Liptzin 2007; Manzoni et al. 2012). However, during rewetting, elevated extractable C can promote SR_pulse_ by delivering a transient surge of readily mineralizable substrates, driving large respiration pulses (Franzluebbers et al. 2000). These contrasting controls were consistent across rhizosphere and non‐rhizosphere soils.

The opposing moisture, temperature, and substrate‐driven controls on SR_overall_ and SR_pulse_ therefore emerge from strong spatial and temporal heterogeneity in soil carbon processing. From a coupled hotspot‐hot moment perspective, these patterns reflect the interaction between rhizosphere hotspots of substrate availability driven by root exudation and extracellular enzyme activity, and rainfall‐driven hot moments that induce rapid, transient activation of microbial metabolism and carbon release (Kuzyakov and Blagodatskaya 2015; Liu et al. 2025; Phillips et al. 2011). Notably, our results identify microbial extracellular enzyme activity as a key predictor of SR_overall_ in the rhizosphere, reinforcing the role of enzyme‐mediated substrate processing within rhizosphere hotspots. Recent frameworks formalize this coupling across spatial scales and ecosystem fluxes (Kannenberg et al. 2024), while empirical evidence shows that precipitation pulses can substantially amplify soil CO_2_ efflux under water‐limited conditions (Zhang, Bilyera, et al. 2023). Together, this coupled hotspot‐hot moment framework provides a mechanistic explanation for the observed decoupling between SR_overall_ and SR_pulse_ under climate extremes.

### 4.5 Advances and Future Directions for Understanding Soil Respiration under Interacting Climate Drivers

Our study addresses several uncertainties surrounding SR responses to interacting climate drivers. Previous work has often focused on drought, warming, or rewetting responses independently, with limited understanding of how these drivers jointly influence baseline and pulse‐driven respiration, whether common environmental controls regulate both processes, and how these responses vary seasonally. By quantifying both SR_overall_ and SR_pulse_ at high temporal resolution, we show that interacting climate drivers simultaneously suppress overall respiration while amplifying rewetting‐induced pulses, and that common environmental and stoichiometric drivers exert contrasting effects on these two components. These findings provide a more integrated understanding of soil carbon fluxes and highlight the need to explicitly represent both overall and pulse‐driven respiration in predictive models. Based on our findings, we outline several key recommendations for advancing long‐term field experiments and SR modeling in the context of increasing climate variability and extremes.

Modeling frameworks should evolve to incorporate the transient nature of the common drivers of SR_overall_ and SR_pulse_ enabling more accurate representation of SR responses to temporal variability and climate extremes. Field studies should employ high‐frequency sampling to capture both baseline and pulse‐driven SR fluctuations, as low‐resolution sampling often misses rapid SR changes following rewetting, especially in dry and warmed conditions. High‐frequency measurements can reveal these short‐term surges, which play a crucial role in annual C budgets (Savage et al. 2009). Monitoring SR at finer temporal scales is essential to capture its true variability under interacting climate drivers. Moreover, moisture sensitivity metrics should be developed in conjunction with Q_10_ to improve predictions of SR under future variation in rainfall, including extreme scenarios: Q_10_, the apparent temperature sensitivity of SR, varies with moisture availability, being highest at intermediate levels. Moreover, moisture sensitivity also co‐determines the magnitude of SR_pulse_. This dual approach will better represent SR responses to variable rainfall conditions.

While our study provides valuable insights, addressing certain limitations might have further strengthened the findings. First, pH and microbial biomass C and N were measured only in the non‐rhizosphere due to limited rhizosphere soil availability and hence were absent in the SEM for rhizosphere soil. Second, the replacement of the top 5 cm of soil to remove legacy effects from prior experiments may have altered the initial soil resource environment and introduced microbial communities that differed from those present in the native surface soil. However, SR measurements commenced approximately 32 months after soil replacement, allowing substantial time for microbial recolonization and ecosystem development under field conditions. Recovery following soil disturbance is generally functionally rapid but compositionally slower, with bacterial‐dominated processes responding more quickly to disturbance than fungal communities, which tend to exhibit slower structural recovery (Jurburg et al. 2017; Shade et al. 2012; Sun et al. 2017). This decoupling between functional and compositional recovery is consistent with microbial resilience frameworks, where functional stability can re‐establish despite ongoing community reassembly over longer timescales (Allison and Martiny 2008; Shade et al. 2012). Importantly, all plots experienced identical soil replacement and subsequent development trajectories, making any residual legacy effects unlikely to systematically bias treatment comparisons. Third, we assessed only the first pulse event in each campaign to simplify the analysis, as our objective was to examine SR responses to rewetting quantity rather than frequency, an approach adopted in controlled laboratory studies (Zhang et al. 2022). We note that, because rainfall events were conducted at night, SR_pulse_ likely reflects nighttime responses and may underestimate the total rewetting effect, but this timing allowed us to isolate the impact of rewetting from temperature‐driven changes in SR. Finally, despite high‐frequency SR measurements during multiple campaigns, data gaps remained. These instances occurred when measurements were discontinued due to plant growth within the soil collars to avoid interference from aboveground respiration. Future studies can overcome these limitations by employing continuous measurement systems in combination with modeling approaches that not only fill data gaps but also quantify process rates, disentangle driver effects, and improve predictive capacity under variable and extreme climatic conditions (Azizi‐Rad et al. 2022; Davidson et al. 2012; Shakoor et al. 2026; Sihi et al. 2018).

## 5 Conclusion

Our study demonstrated that drought and warming consistently suppressed SR_overall_, with the nature of their interactions varying seasonally, while simultaneously amplifying rewetting‐induced SR_pulse_. Pulse magnitude and post‐pulse decline in SR were primarily governed by moisture dynamics and temperature. These treatments also enhanced SR's responsiveness to soil moisture and amplified its temperature sensitivity under wetter conditions. Furthermore, temperature, moisture, and substrate availability (extractable C:N ratio) were key drivers of both SR_overall_ and SR_pulse_, and while these relationships were consistent across rhizosphere and non‐rhizosphere zones, the drivers influenced the two components in opposing directions. These findings highlight contrasting controls on baseline and pulse‐driven respiration, emphasizing the importance of considering both processes to improve model representations of SR and enable more accurate predictions of soil C dynamics under future interacting climate drivers.

## Author Contributions

**Pankaj Tiwari:** conceptualization, methodology, data curation, writing – original draft, writing – review and editing, formal analysis, software, visualization, investigation. **Elise Pendall:** conceptualization, funding acquisition, writing – review and editing, supervision, data curation, investigation, methodology. **Nicholas Wright‐Osment:** data curation, investigation, writing – review and editing. **Nor Azizah Kusai:** data curation, investigation, writing – review and editing. **Manjunatha H. Chandregowda:** conceptualization, writing – review and editing. **Awais Shakoor:** investigation, writing – review and editing. **Debjani Sihi:** writing – review and editing, funding acquisition. **Sally A. Power:** funding acquisition, writing – review and editing. **Eric A. Davidson:** funding acquisition, writing – review and editing. **Catriona A. Macdonald:** conceptualization, methodology, data curation, investigation, supervision, funding acquisition, writing – review and editing.

## Funding

This study was supported by the Australian Research Council (ARCDP220102039), the Australian Government's National Collaborative Research Infrastructure Strategy via the Terrestrial Ecosystem Research Network, Meat and Livestock Australia's Donor Company (P.PSH.2009), and the Commonwealth through an Australian Government Research Training Program Scholarship (DOI: https://doi.org/10.82133/C42F‐K220).

## Conflicts of Interest

The authors declare no conflicts of interest.

## Supporting information


**Figure S1:** Experimental shelter at the PAstures and Climate Extremes (PACE) facility (Richmond, New South Wales, Australia, −33.60972, 150.73833, 25 m asl), illustrating the infrastructure used for field‐based climate manipulation.
**Figure S2:** Simulated rainfall in Dry and Wet treatment across the study period.
**Figure S3:** (A) Soil respiration‐temperature and (B) soil respiration‐moisture relationships.
**Figure S4:** Effects of drought and warming on aboveground and belowground biomass.
**Figure S5:** Effects of drought and warming on soil physicochemical parameters in non‐rhizosphere (hatched) and rhizosphere (dotted) in 0–5 cm and 5–10 cm depth.
**Figure S6:** Effects of drought and warming on microbial extracellular enzyme activity in non‐rhizosphere and rhizosphere in 0–5 cm and 5–10 cm depth.
**Figure S7:** Relationship between soil respiration (SR) and aboveground biomass (AGB) across different treatments.
**Figure S8:** Soil water content (SWC) response to rewetting.
**Table S1:** Seasonal rainfall and total rainfall events in Wet and Dry treatments during 2024.
**Table S2:** Drought and Warming effects on SR_overall_, soil microclimate, aboveground biomass and rewetting response parameters.

## Data Availability

The data that support the findings of this study are available at https://doi.org/10.6084/m9.figshare.30154363.v2 (Tiwari et al. 2026 Main Data_ Drought and warming‐induced drying suppress soil respiration but amplify rewetting‐induced pulses in a temperate pasture.xlsx, 2026).

## References

[gcb71005-bib-0001] AghaKouchak, A. , F. Chiang , L. S. Huning , et al. 2020. “Climate Extremes and Compound Hazards in a Warming World.” Annual Review of Earth and Planetary Sciences 48, no. 1: 519–548. 10.1146/annurev-earth-071719-055228.

[gcb71005-bib-0002] Ahern, C. R. , D. E. Baker , and R. L. Aitken . 1995. “Models for Relating pH Measurements in Water and Calcium Chloride for a Wide Range of pH, Soil Types and Depths.” In Plant‐Soil Interactions at Low pH: Principles and Management, edited by R. A. Date , N. J. Grundon , G. E. Rayment , and M. E. Probert , 99–104. Springer Netherlands. 10.1007/978-94-011-0221-6_13.

[gcb71005-bib-0003] Allison, S. D. , and J. B. H. Martiny . 2008. “Resistance, Resilience, and Redundancy in Microbial Communities.” Proceedings of the National Academy of Sciences 105, no. supplement_1: 11512–11519. 10.1073/pnas.0801925105. PMC2556421 18695234

[gcb71005-bib-0004] Allison, S. D. , and P. M. Vitousek . 2004. “Extracellular Enzyme Activities and Carbon Chemistry as Drivers of Tropical Plant Litter Decomposition.” Biotropica 36, no. 3: 285–296. 10.1111/j.1744-7429.2004.tb00321.x.

[gcb71005-bib-0005] Allison, S. D. , and P. M. Vitousek . 2005. “Responses of Extracellular Enzymes to Simple and Complex Nutrient Inputs.” Soil Biology and Biochemistry 37, no. 5: 937–944. 10.1016/j.soilbio.2004.09.014.

[gcb71005-bib-0006] Anthony, T. L. , and W. L. Silver . 2023. “Hot Spots and Hot Moments of Greenhouse Gas Emissions in Agricultural Peatlands.” Biogeochemistry 167, no. 4: 461–477. 10.1007/s10533-023-01095-y.

[gcb71005-bib-0007] Appuhn, A. , R. G. Joergensen , M. Raubuch , E. Scheller , and B. Wilke . 2004. “The Automated Determination of Glucosamine, Galactosamine, Muramic Acid, and Mannosamine in Soil and Root Hydrolysates by HPLC.” Journal of Plant Nutrition and Soil Science 167, no. 1: 17–21. 10.1002/jpln.200321302.

[gcb71005-bib-0008] Azizi‐Rad, M. , G. Guggenberger , Y. Ma , and C. A. Sierra . 2022. “Sensitivity of Soil Respiration Rate With Respect to Temperature, Moisture and Oxygen Under Freezing and Thawing.” Soil Biology and Biochemistry 165: 108488. 10.1016/j.soilbio.2021.108488.

[gcb71005-bib-0009] Barnard, R. L. , S. J. Blazewicz , and M. K. Firestone . 2020. “Rewetting of Soil: Revisiting the Origin of Soil CO_2_ Emissions.” Soil Biology and Biochemistry 147: 107819. 10.1016/j.soilbio.2020.107819.

[gcb71005-bib-0010] Bates, D. , M. Mächler , B. Bolker , and S. Walker . 2015. “Fitting Linear Mixed‐Effects Models Using lme4.” Journal of Statistical Software 67: 1–48. 10.18637/jss.v067.i01.

[gcb71005-bib-0011] Bell, C. W. , B. E. Fricks , J. D. Rocca , J. M. Steinweg , S. K. McMahon , and M. D. Wallenstein . 2013. “High‐Throughput Fluorometric Measurement of Potential Soil Extracellular Enzyme Activities.” JoVE (Journal of Visualized Experiments) 81: e50961. 10.3791/50961. PMC3991303 24299913

[gcb71005-bib-0012] Bian, H. , C. Li , J. Zhu , et al. 2022. “Soil Moisture Affects the Rapid Response of Microbes to Labile Organic C Addition.” Frontiers in Ecology and Evolution 10: 857185. 10.3389/fevo.2022.857185.

[gcb71005-bib-0013] Birch, H. F. 1958. “The Effect of Soil Drying on Humus Decomposition and Nitrogen Availability.” Plant and Soil 10, no. 1: 9–31. 10.1007/BF01343734.

[gcb71005-bib-0014] Bond‐Lamberty, B. , A. Ballantyne , E. Berryman , et al. 2024. “Twenty Years of Progress, Challenges, and Opportunities in Measuring and Understanding Soil Respiration.” Journal of Geophysical Research, Biogeosciences 129, no. 2: e2023JG007637. 10.1029/2023JG007637.

[gcb71005-bib-0015] Borken, W. , and E. Matzner . 2009. “Reappraisal of Drying and Wetting Effects on C and N Mineralization and Fluxes in Soils.” Global Change Biology 15, no. 4: 808–824. 10.1111/j.1365-2486.2008.01681.x.

[gcb71005-bib-0016] Bouskill, N. J. , S. S. Chacon , D. F. Cusack , et al. 2025. “Climate History Modulates Stress Responses of Common Soil Bacteria Under Experimental Drought.” ISME Journal 19, no. 1: wraf075. 10.1093/ismejo/wraf075. 40247716 PMC12085270

[gcb71005-bib-0017] Bouskill, N. J. , T. E. Wood , R. Baran , et al. 2016. “Belowground Response to Drought in a Tropical Forest Soil. I. Changes in Microbial Functional Potential and Metabolism.” Frontiers in Microbiology 7: 525. 10.3389/fmicb.2016.00525. 27148214 PMC4837414

[gcb71005-bib-0018] Bureau of Meteorology . 2025. “Australia's Official Weather Forecasts & Weather Radar—Bureau of Meteorology.” http://www.bom.gov.au/.

[gcb71005-bib-0019] Chandregowda, M. H. , M. G. Tjoelker , E. Pendall , H. Zhang , A. C. Churchill , and S. A. Power . 2023. “Belowground Carbon Allocation, Root Trait Plasticity, and Productivity During Drought and Warming in a Pasture Grass.” Journal of Experimental Botany 74, no. 6: 2127–2145. 10.1093/jxb/erad021. 36640126 PMC10084810

[gcb71005-bib-0020] Churchill, A. C. , H. Zhang , K. J. Fuller , et al. 2022. “Pastures and Climate Extremes: Impacts of Cool Season Warming and Drought on the Productivity of Key Pasture Species in a Field Experiment.” Frontiers in Plant Science 13: 836968. 10.3389/fpls.2022.836968. 35321443 PMC8937038

[gcb71005-bib-0021] Cleveland, C. C. , and D. Liptzin . 2007. “C:N:P Stoichiometry in Soil: Is There a ‘Redfield Ratio’ for the Microbial Biomass?” Biogeochemistry 85, no. 3: 235–252. 10.1007/s10533-007-9132-0.

[gcb71005-bib-0022] Cook, F. J. , and V. A. Orchard . 2008. “Relationships Between Soil Respiration and Soil Moisture.” Soil Biology and Biochemistry 40, no. 5: 1013–1018. 10.1016/j.soilbio.2007.12.012.

[gcb71005-bib-0023] Crain, C. M. , K. Kroeker , and B. S. Halpern . 2008. “Interactive and Cumulative Effects of Multiple Human Stressors in Marine Systems.” Ecology Letters 11, no. 12: 1304–1315. 10.1111/j.1461-0248.2008.01253.x. 19046359

[gcb71005-bib-0024] Davidson, E. A. , and I. A. Janssens . 2006. “Temperature Sensitivity of Soil Carbon Decomposition and Feedbacks to Climate Change.” Nature 440, no. 7081: 165–173. 10.1038/nature04514. 16525463

[gcb71005-bib-0025] Davidson, E. A. , S. Samanta , S. S. Caramori , and K. Savage . 2012. “The Dual Arrhenius and Michaelis–Menten Kinetics Model for Decomposition of Soil Organic Matter at Hourly to Seasonal Time Scales.” Global Change Biology 18, no. 1: 371–384. 10.1111/j.1365-2486.2011.02546.x.

[gcb71005-bib-0026] Denissen, J. M. C. , A. J. Teuling , S. Koirala , et al. 2024. “Intensified Future Heat Extremes Linked With Increasing Ecosystem Water Limitation.” Earth System Dynamics 15, no. 3: 717–734. 10.5194/esd-15-717-2024.

[gcb71005-bib-0027] Dey, R. , S. C. Lewis , J. M. Arblaster , and N. J. Abram . 2019. “A Review of Past and Projected Changes in Australia's Rainfall.” WIREs Climate Change 10, no. 3: e577. 10.1002/wcc.577.

[gcb71005-bib-0028] Dinh, M.‐V. , A. Guhr , A. R. Weig , and E. Matzner . 2018. “Drying and Rewetting of Forest Floors: Dynamics of Soluble Phosphorus, Microbial Biomass‐Phosphorus, and the Composition of Microbial Communities.” Biology and Fertility of Soils 54, no. 6: 761–768. 10.1007/s00374-018-1300-y.

[gcb71005-bib-0029] Dong, H. , S. Zhang , J. Lin , and B. Zhu . 2021. “Responses of Soil Microbial Biomass Carbon and Dissolved Organic Carbon to Drying‐Rewetting Cycles: A Meta‐Analysis.” Catena 207: 105610. 10.1016/j.catena.2021.105610.

[gcb71005-bib-0030] Du, Y. , J. Mohan , P. Frankson , G. Franke , Z. Chen , and D. Sihi . 2025. “Decoding the Hidden Mechanisms of Soil Carbon Cycling in Response to Climate Change in a Substrate‐Limited Forested Ecosystem.” Biogeochemistry 168, no. 5: 74. 10.1007/s10533-025-01265-0.

[gcb71005-bib-0031] FAO . 2023. The State of Food Security and Nutrition in the World 2023. FAO, IFAD, UNICEF, WFP, WHO. 10.4060/cc3017en.

[gcb71005-bib-0032] Fernandez‐Bou, A. S. , D. Dierick , M. F. Allen , and T. C. Harmon . 2020. “Precipitation‐Drainage Cycles Lead to Hot Moments in Soil Carbon Dioxide Dynamics in a Neotropical Wet Forest.” Global Change Biology 26, no. 9: 5303–5319. 10.1111/gcb.15194. 32458420

[gcb71005-bib-0033] Franzluebbers, A. J. , R. L. Haney , C. W. Honeycutt , H. H. Schomberg , and F. M. Hons . 2000. “Flush of Carbon Dioxide Following Rewetting of Dried Soil Relates to Active Organic Pools.” Soil Science Society of America Journal 64, no. 2: 613–623. 10.2136/sssaj2000.642613x.

[gcb71005-bib-0034] Friedlingstein, P. , M. O'Sullivan , M. W. Jones , et al. 2025. “Global Carbon Budget 2024.” Earth System Science Data 17, no. 3: 965–1039. 10.5194/essd-17-965-2025.

[gcb71005-bib-0035] Groffman, P. M. , K. Butterbach‐Bahl , R. W. Fulweiler , et al. 2009. “Challenges to Incorporating Spatially and Temporally Explicit Phenomena (Hotspots and Hot Moments) in Denitrification Models.” Biogeochemistry 93, no. 1: 49–77. 10.1007/s10533-008-9277-5.

[gcb71005-bib-0036] Guan, C. , N. Chen , L. Qiao , X. Ma , and C. Zhao . 2023. “Biocrusts Regulate the Effect of Rainfall Pulses on Soil Respiration at Different Temporal Scales on the Loess Plateau.” Soil Biology and Biochemistry 180: 109018. 10.1016/j.soilbio.2023.109018.

[gcb71005-bib-0037] Haney, R. L. , A. J. Franzluebbers , V. L. Jin , et al. 2012. “Soil Organic C:N vs. Water‐Extractable Organic C:N.” Open Journal of Soil Science 2, no. 3: 3. 10.4236/ojss.2012.23032.

[gcb71005-bib-0038] He, H. , T. Zha , and J. Tan . 2024. “Soil‐Moisture‐Dependent Temperature Sensitivity of Soil Respiration in a Poplar Plantation in Northern China.” Forests 15, no. 8: 1466. 10.3390/f15081466.

[gcb71005-bib-0039] He, Y. , X. Zhou , Z. Jia , et al. 2023. “Apparent Thermal Acclimation of Soil Heterotrophic Respiration Mainly Mediated by Substrate Availability.” Global Change Biology 29, no. 4: 1178–1187. 10.1111/gcb.16523. 36371668

[gcb71005-bib-0040] Hoover, D. L. , A. K. Knapp , and M. D. Smith . 2016. “The Immediate and Prolonged Effects of Climate Extremes on Soil Respiration in a Mesic Grassland.” Journal of Geophysical Research: Biogeosciences 121, no. 4: 1034–1044. 10.1002/2015JG003256.

[gcb71005-bib-0041] Houghton, R. A. , and A. A. Nassikas . 2017. “Global and Regional Fluxes of Carbon From Land Use and Land Cover Change 1850–2015.” Global Biogeochemical Cycles 31, no. 3: 456–472. 10.1002/2016GB005546.

[gcb71005-bib-0042] Jarvis, P. , A. Rey , C. Petsikos , et al. 2007. “Drying and Wetting of Mediterranean Soils Stimulates Decomposition and Carbon Dioxide Emission: The ‘Birch Effect’.” Tree Physiology 27, no. 7: 929–940. 10.1093/treephys/27.7.929. 17403645

[gcb71005-bib-0043] Joergensen, R. G. , and T. Mueller . 1996. “The Fumigation‐Extraction Method to Estimate Soil Microbial Biomass: Calibration of the *k*EN Value.” Soil Biology and Biochemistry 28, no. 1: 33–37. 10.1016/0038-0717(95)00101-8.

[gcb71005-bib-0044] Jones, D. L. , A. Hodge , and Y. Kuzyakov . 2004. “Plant and Mycorrhizal Regulation of Rhizodeposition.” New Phytologist 163, no. 3: 459–480. 10.1111/j.1469-8137.2004.01130.x. 33873745

[gcb71005-bib-0045] Jurburg, S. D. , I. Nunes , J. C. Stegen , et al. 2017. “Autogenic Succession and Deterministic Recovery Following Disturbance in Soil Bacterial Communities.” Scientific Reports 7, no. 1: 45691. 10.1038/srep45691. 28383027 PMC5382530

[gcb71005-bib-0046] Kannenberg, S. A. , W. R. L. Anderegg , M. L. Barnes , M. P. Dannenberg , and A. K. Knapp . 2024. “Dominant Role of Soil Moisture in Mediating Carbon and Water Fluxes in Dryland Ecosystems.” Nature Geoscience 17, no. 1: 38–43. 10.1038/s41561-023-01351-8.

[gcb71005-bib-0047] Kite, I. L. , S. A. Power , R. G. Meyer , et al. 2025. “Evaluating Nutritional Quality and Methane Production From Fermentation of Pasture Forages Grown Under Current and Future Climate Conditions Using Near‐Infrared Spectroscopy.” Crop & Pasture Science 76, no. 8: CP24374. 10.1071/CP24374.

[gcb71005-bib-0048] Klein, J. A. , J. Harte , and X.‐Q. Zhao . 2004. “Experimental Warming Causes Large and Rapid Species Loss, Dampened by Simulated Grazing, on the Tibetan Plateau.” Ecology Letters 7, no. 12: 1170–1179. 10.1111/j.1461-0248.2004.00677.x.

[gcb71005-bib-0049] Kline, R. B. 2016. Principles and Practice of Structural Equation Modeling. 4th ed (pp. xvii, 534). Guilford Press.

[gcb71005-bib-0050] Kurganova, I. , V. Lopes de Gerenyu , D. Khoroshaev , T. Myakshina , D. Sapronov , and V. Zhmurin . 2022. “Temperature Sensitivity of Soil Respiration in Two Temperate Forest Ecosystems: The Synthesis of a 24‐Year Continuous Observation.” Forests 13, no. 9: 9. 10.3390/f13091374.

[gcb71005-bib-0051] Kuznetsova, A. , P. B. Brockhoff , and R. H. B. Christensen . 2017. “lmerTest Package: Tests in Linear Mixed Effects Models.” Journal of Statistical Software 82: 1–26. 10.18637/jss.v082.i13.

[gcb71005-bib-0052] Kuzyakov, Y. , and E. Blagodatskaya . 2015. “Microbial Hotspots and Hot Moments in Soil: Concept & Review.” Soil Biology and Biochemistry 83: 184–199. 10.1016/j.soilbio.2015.01.025.

[gcb71005-bib-0053] Lado‐Monserrat, L. , C. Lull , I. Bautista , A. Lidón , and R. Herrera . 2014. “Soil Moisture Increment as a Controlling Variable of the ‘Birch Effect’. Interactions With the Pre‐Wetting Soil Moisture and Litter Addition.” Plant and Soil 379, no. 1: 21–34. 10.1007/s11104-014-2037-5.

[gcb71005-bib-0054] Lee, J. M. , A. J. Clark , and J. R. Roche . 2013. “Climate‐Change Effects and Adaptation Options for Temperate Pasture‐Based Dairy Farming Systems: A Review.” Grass and Forage Science 68, no. 4: 485–503. 10.1111/gfs.12039.

[gcb71005-bib-0055] Lenth, R. V. , B. Banfai , B. Bolker , et al. 2025. “emmeans: Estimated Marginal Means, aka Least‐Squares Means (Version 1.11.2) [Computer Software].” https://cran.r‐project.org/web/packages/emmeans/index.html.

[gcb71005-bib-0056] Li, J. , J. Zhang , T. Ma , et al. 2023. “Responses of Soil Respiration to the Interactive Effects of Warming and Drought in Alfalfa Grassland on the Loess Plateau.” Agronomy 13, no. 12: 12. 10.3390/agronomy13122992.

[gcb71005-bib-0057] Li, X. , M. Pallandt , D. Naidu , J. Rousk , G. Hugelius , and S. Manzoni . 2025. “Validating Laboratory Predictions of Soil Rewetting Respiration Pulses Using Field Data.” Biogeosciences 22, no. 11: 2691–2705. 10.5194/bg-22-2691-2025.

[gcb71005-bib-0058] Li, X. , Y. Yan , and L. Fu . 2021. “Effects of Rainfall Manipulation on Ecosystem Respiration and Soil Respiration in an Alpine Steppe in Northern Tibet Plateau.” Frontiers in Ecology and Evolution 9: 708761. 10.3389/fevo.2021.708761.

[gcb71005-bib-0059] Liang, G. , S. C. Reed , J. M. Stark , and B. G. Waring . 2023. “Unraveling Mechanisms Underlying Effects of Wetting–Drying Cycles on Soil Respiration in a Dryland.” Biogeochemistry 166, no. 1: 23–37. 10.1007/s10533-023-01085-0.

[gcb71005-bib-0060] Liang, G. , A. Stefanski , W. C. Eddy , et al. 2024. “Soil Respiration Response to Decade‐Long Warming Modulated by Soil Moisture in a Boreal Forest.” Nature Geoscience 17, no. 9: 905–911. 10.1038/s41561-024-01512-3.

[gcb71005-bib-0061] Liu, J. , J. Hu , H. Liu , and K. Han . 2024. “Global Soil Respiration Estimation Based on Ecological Big Data and Machine Learning Model.” Scientific Reports 14, no. 1: 13231. 10.1038/s41598-024-64235-w. 38853165 PMC11163009

[gcb71005-bib-0062] Liu, L. , M. Estiarte , P. Bengtson , et al. 2022. “Drought Legacies on Soil Respiration and Microbial Community in a Mediterranean Forest Soil Under Different Soil Moisture and Carbon Inputs.” Geoderma 405: 115425. 10.1016/j.geoderma.2021.115425.

[gcb71005-bib-0063] Liu, L. , X. Wang , M. J. Lajeunesse , et al. 2016. “A Cross‐Biome Synthesis of Soil Respiration and Its Determinants Under Simulated Precipitation Changes.” Global Change Biology 22, no. 4: 1394–1405. 10.1111/gcb.13156. 26554753

[gcb71005-bib-0064] Liu, Z. , Y. Kuzyakov , J. Fang , et al. 2025. “Hotspots of Enzyme Activities Reflect Micro‐Scale Heterogeneity in Nutrient Mobilization in Paddy Soils.” Plant and Soil 513, no. 1: 1433–1450. 10.1007/s11104-025-07265-1.

[gcb71005-bib-0065] Lorenz, K. , and R. Lal . 2018. “Carbon Sequestration in Grassland Soils.” In Carbon Sequestration in Agricultural Ecosystems, edited by K. Lorenz and R. Lal , 175–209. Springer International Publishing. 10.1007/978-3-319-92318-5_4.

[gcb71005-bib-0066] Lu, Y. , H. Liu , X. Zhou , et al. 2025. “Response Characteristics of Bulk Soil, Rhizosphere, and Root Endophytic Microbiota in Desert Ephemeral Plants to Increased Precipitation.” Plant and Soil 513: 3073–3095. 10.1007/s11104-025-07374-x.

[gcb71005-bib-0067] Manzoni, S. , A. Chakrawal , T. Fischer , J. P. Schimel , A. Porporato , and G. Vico . 2020. “Rainfall Intensification Increases the Contribution of Rewetting Pulses to Soil Heterotrophic Respiration.” Biogeosciences 17, no. 15: 4007–4023. 10.5194/bg-17-4007-2020.

[gcb71005-bib-0068] Manzoni, S. , P. Taylor , A. Richter , A. Porporato , and G. I. Ågren . 2012. “Environmental and Stoichiometric Controls on Microbial Carbon‐Use Efficiency in Soils.” New Phytologist 196, no. 1: 79–91. 10.1111/j.1469-8137.2012.04225.x. 22924405

[gcb71005-bib-0069] McCaskill, M. R. , M. C. Raeside , S. G. Clark , C. MacDonald , B. Clark , and D. L. Partington . 2016. “Pasture Mixes With Lucerne ( *Medicago sativa* ) Increase Yields and Water‐Use Efficiencies Over Traditional Pastures Based on Subterranean Clover ( *Trifolium subterraneum* ).” Crop and Pasture Science 67, no. 1: 69–80. 10.1071/CP14179.

[gcb71005-bib-0070] Meeran, K. , J. Ingrisch , D. Reinthaler , et al. 2021. “Warming and Elevated CO_2_ Intensify Drought and Recovery Responses of Grassland Carbon Allocation to Soil Respiration.” Global Change Biology 27, no. 14: 3230–3243. 10.1111/gcb.15628. 33811716

[gcb71005-bib-0071] Metz, E.‐M. , S. N. Vardag , S. Basu , et al. 2023. “Soil Respiration–Driven CO_2_ Pulses Dominate Australia's Flux Variability.” Science 379, no. 6639: 1332–1335. 10.1126/science.add7833. 36996200

[gcb71005-bib-0072] Morris, K. A. , S. Hornum , R. Crystal‐Ornelas , S. C. Pennington , and B. Bond‐Lamberty . 2022. “Soil Respiration Response to Simulated Precipitation Change Depends on Ecosystem Type and Study Duration.” Journal of Geophysical Research: Biogeosciences 127, no. 11: e2022JG006887. 10.1029/2022JG006887.

[gcb71005-bib-0073] Moyano, F. E. , S. Manzoni , and C. Chenu . 2013. “Responses of Soil Heterotrophic Respiration to Moisture Availability: An Exploration of Processes and Models.” Soil Biology and Biochemistry 59: 72–85.

[gcb71005-bib-0074] Najera, F. , M. A. Dippold , J. Boy , et al. 2020. “Effects of Drying/Rewetting on Soil Aggregate Dynamics and Implications for Organic Matter Turnover.” Biology and Fertility of Soils 56, no. 7: 893–905. 10.1007/s00374-020-01469-6.

[gcb71005-bib-0075] Navarro‐García, F. , M. Á. Casermeiro , and J. P. Schimel . 2012. “When Structure Means Conservation: Effect of Aggregate Structure in Controlling Microbial Responses to Rewetting Events.” Soil Biology and Biochemistry 44, no. 1: 1–8. 10.1016/j.soilbio.2011.09.019.

[gcb71005-bib-0076] Nguyen, N. B. , M. Migliavacca , M. Bassiouni , et al. 2025. “Widespread Underestimation of Rain‐Induced Soil Carbon Emissions From Global Drylands.” Nature Geoscience 18: 1–8. 10.1038/s41561-025-01754-9. PMC12422978 40949426

[gcb71005-bib-0077] Niu, S. , M. Wu , Y. Han , J. Xia , L. Li , and S. Wan . 2008. “Water‐Mediated Responses of Ecosystem Carbon Fluxes to Climatic Change in a Temperate Steppe.” New Phytologist 177, no. 1: 209–219. 10.1111/j.1469-8137.2007.02237.x. 17944829

[gcb71005-bib-0078] Pallandt, M. , B. Ahrens , S. Koirala , et al. 2022. “Vertically Divergent Responses of SOC Decomposition to Soil Moisture in a Changing Climate.” Journal of Geophysical Research: Biogeosciences 127, no. 2: e2021JG006684. 10.1029/2021JG006684.

[gcb71005-bib-0079] Patel, K. F. , A. Myers‐Pigg , B. Bond‐Lamberty , et al. 2021. “Soil Carbon Dynamics During Drying vs. Rewetting: Importance of Antecedent Moisture Conditions.” Soil Biology and Biochemistry 156: 108165. 10.1016/j.soilbio.2021.108165.

[gcb71005-bib-0080] Pearce, K. B. , P. N. Holper , M. Hopkins , et al. 2007. “CSIRO Research Publications Repository—Climate Change in Australia.” Technical Report, p. 2007.

[gcb71005-bib-0081] Peng, S. , S. Piao , T. Wang , J. Sun , and Z. Shen . 2009. “Temperature Sensitivity of Soil Respiration in Different Ecosystems in China.” Soil Biology and Biochemistry, Science Goes Underground in China 41, no. 5: 1008–1014. 10.1016/j.soilbio.2008.10.023.

[gcb71005-bib-0082] Phillips, R. P. , and T. J. Fahey . 2006. “Tree Species and Mycorrhizal Associations Influence the Magnitude of Rhizosphere Effects.” Ecology 87, no. 5: 1302–1313. 10.1890/0012-9658(2006)87[1302:TSAMAI]2.0.CO;2. 16761608

[gcb71005-bib-0083] Phillips, R. P. , A. C. Finzi , and E. S. Bernhardt . 2011. “Enhanced Root Exudation Induces Microbial Feedbacks to N Cycling in a Pine Forest Under Long‐Term CO_2_ Fumigation.” Ecology Letters 14, no. 2: 187–194. 10.1111/j.1461-0248.2010.01570.x. 21176050

[gcb71005-bib-0084] Piggott, J. J. , C. R. Townsend , and C. D. Matthaei . 2015. “Reconceptualizing Synergism and Antagonism Among Multiple Stressors.” Ecology and Evolution 5, no. 7: 1538–1547. 10.1002/ece3.1465. 25897392 PMC4395182

[gcb71005-bib-0085] Pinheiro, J. C. , and D. M. Bates , eds. 2000. “Fitting Nonlinear Mixed‐Effects Models.” In Mixed‐Effects Models in S and S‐PLUS, 337–421. Springer. 10.1007/0-387-22747-4_8.

[gcb71005-bib-0086] R Core Team . 2026. “R: The R Project for Statistical Computing.” https://www.r‐project.org/.

[gcb71005-bib-0087] Reichstein, M. , M. Bahn , P. Ciais , et al. 2013. “Climate Extremes and the Carbon Cycle.” Nature 500, no. 7462: 7462. 10.1038/nature12350. 23955228

[gcb71005-bib-0088] Reinthaler, D. , E. Harris , E. M. Pötsch , et al. 2021. “Responses of Grassland Soil CO_2_ Production and Fluxes to Drought Are Shifted in a Warmer Climate Under Elevated CO_2_ .” Soil Biology and Biochemistry 163: 108436. 10.1016/j.soilbio.2021.108436.

[gcb71005-bib-0089] Rosinger, C. , J. Rousk , M. Bonkowski , J. Rethemeyer , and A. Jaeschke . 2023. “Rewetting the Hyper‐Arid Atacama Desert Soil Reactivates a Carbon‐Starved Microbial Decomposer Community and Also Triggers Archaeal Metabolism.” Science of the Total Environment 892: 164785. 10.1016/j.scitotenv.2023.164785. 37302588

[gcb71005-bib-0090] Savage, K. , E. A. Davidson , A. D. Richardson , and D. Y. Hollinger . 2009. “Three Scales of Temporal Resolution From Automated Soil Respiration Measurements. Agricultural and Forest Meteorology, Special Section on Water and Carbon Dynamics in Selected Ecosystems in China.” 149, no. 11: 2012–2021. 10.1016/j.agrformet.2009.07.008.

[gcb71005-bib-0091] Schermelleh‐Engel, K. , H. Moosbrugger , and H. Müller . 2003. “Evaluating the Fit of Structural Equation Models: Tests of Significance and Descriptive Goodness‐of‐Fit Measures.” Methods of Psychological Research 8, no. 2: 23–74.

[gcb71005-bib-0092] Schimel, J. P. 2018. “Life in Dry Soils: Effects of Drought on Soil Microbial Communities and Processes.” Annual Review of Ecology, Evolution, and Systematics 49, no. 1: 409–432. 10.1146/annurev-ecolsys-110617-062614.

[gcb71005-bib-0093] Schindlbacher, A. , S. Wunderlich , W. Borken , B. Kitzler , S. Zechmeister‐Boltenstern , and R. Jandl . 2012. “Soil Respiration Under Climate Change: Prolonged Summer Drought Offsets Soil Warming Effects.” Global Change Biology 18, no. 7: 2270–2279. 10.1111/j.1365-2486.2012.02696.x.

[gcb71005-bib-0094] Schmidt, I. , S. Reinsch , and C. Christiansen . 2021. “2.2.1 Soil Microbial Biomass—C, N, and P—ClimEx Handbook.” https://climexhandbook.w.uib.no/2019/11/06/soil‐microbial‐biomass‐c‐n‐and‐p/?utm_source=chatgpt.com.

[gcb71005-bib-0095] Seó, H. L. S. , L. C. P. Machado Filho , and D. Brugnara . 2017. “Rationally Managed Pastures Stock More Carbon Than no‐Tillage Fields.” Frontiers in Environmental Science 5: 87. 10.3389/fenvs.2017.00087.

[gcb71005-bib-0096] Shade, A. , H. Peter , S. D. Allison , et al. 2012. “Fundamentals of Microbial Community Resistance and Resilience.” Frontiers in Microbiology 3: 417. 10.3389/fmicb.2012.00417. 23267351 PMC3525951

[gcb71005-bib-0097] Shakoor, A. , P. Tiwari , N. Wright‐Osment , et al. 2026. “Improving Prediction of Heterotrophic Respiration in Response to Altered Precipitation.” Journal of Geophysical Research: Biogeosciences 131, no. 2: e2025JG009411. 10.1029/2025JG009411.

[gcb71005-bib-0098] Sherwood, S. C. , M. J. Webb , J. D. Annan , et al. 2020. “An Assessment of Earth's Climate Sensitivity Using Multiple Lines of Evidence.” Reviews of Geophysics 58, no. 4: e2019RG000678. 10.1029/2019RG000678. PMC7524012 33015673

[gcb71005-bib-0099] Sierra, C. A. , S. E. Trumbore , E. A. Davidson , S. Vicca , and I. Janssens . 2015. “Sensitivity of Decomposition Rates of Soil Organic Matter With Respect to Simultaneous Changes in Temperature and Moisture.” Journal of Advances in Modeling Earth Systems 7, no. 1: 335–356. 10.1002/2014MS000358.

[gcb71005-bib-0100] Sihi, D. , E. A. Davidson , M. Chen , et al. 2018. “Merging a Mechanistic Enzymatic Model of Soil Heterotrophic Respiration Into an Ecosystem Model in Two AmeriFlux Sites of Northeastern USA.” Agricultural and Forest Meteorology 252: 155–166. 10.1016/j.agrformet.2018.01.026.

[gcb71005-bib-0101] Slessarev, E. W. , and J. P. Schimel . 2020. “Partitioning Sources of CO_2_ Emission After Soil Wetting Using High‐Resolution Observations and Minimal Models.” Soil Biology and Biochemistry 143: 107753. 10.1016/j.soilbio.2020.107753.

[gcb71005-bib-0102] Stark, J. M. , and M. K. Firestone . 1995. “Mechanisms for Soil Moisture Effects on Activity of Nitrifying Bacteria.” Applied and Environmental Microbiology 61, no. 1: 218–221. 10.1128/aem.61.1.218-221.1995. 16534906 PMC1388328

[gcb71005-bib-0103] Stokes, C. J. , A. Ash , and S. M. Howden . 2008. “Climate Change Impacts on Australian Rangelands.” Rangelands 30, no. 3: 40–45.

[gcb71005-bib-0104] Sun, Q. , W. S. Meyer , G. R. Koerber , and P. Marschner . 2015. “Response of Respiration and Nutrient Availability to Drying and Rewetting in Soil From a Semi‐Arid Woodland Depends on Vegetation Patch and a Recent Wildfire.” Biogeosciences 12, no. 16: 5093–5101. 10.5194/bg-12-5093-2015.

[gcb71005-bib-0105] Sun, S. , S. Li , B. N. Avera , B. D. Strahm , and B. D. Badgley . 2017. “Soil Bacterial and Fungal Communities Show Distinct Recovery Patterns During Forest Ecosystem Restoration.” Applied and Environmental Microbiology 83, no. 14: e00966‐17. 10.1128/AEM.00966-17. 28476769 PMC5494632

[gcb71005-bib-0106] Suseela, V. , R. T. Conant , M. D. Wallenstein , and J. S. Dukes . 2012. “Warming and Drought Alter Q10 of Soil Respiration in Temperate Grasslands.” Global Change Biology 18, no. 1: 285–296. 10.1111/j.1365-2486.2011.02516.x.

[gcb71005-bib-0107] Tecon, R. , and D. Or . 2017. “Biophysical Processes Supporting the Diversity of Microbial Life in Soil.” FEMS Microbiology Reviews 41, no. 5: 599–623. 10.1093/femsre/fux039. 28961933 PMC5812502

[gcb71005-bib-0108] Timbal, B. 2004. “Southwest Australia Past and Future Rainfall Trends.” Climate Research 26: 233–249. 10.3354/cr026233.

[gcb71005-bib-0109] Tiwari, P. , P. Bhattacharya , G. S. Rawat , I. D. Rai , and G. Talukdar . 2021. “Experimental Warming Increases Ecosystem Respiration by Increasing Above‐Ground Respiration in Alpine Meadows of Western Himalaya.” Scientific Reports 11, no. 1: 2640. 10.1038/s41598-021-82065-y. 33514838 PMC7846769

[gcb71005-bib-0110] Tiwari, P. , P. Bhattacharya , G. S. Rawat , and G. Talukdar . 2021. “Equilibrium in Soil Respiration Across a Climosequence Indicates Its Resilience to Climate Change in a Glaciated Valley, Western Himalaya.” Scientific Reports 11, no. 1: 23038. 10.1038/s41598-021-02199-x. 34845254 PMC8630114

[gcb71005-bib-0111] Tripathy, K. P. , S. Mukherjee , A. K. Mishra , M. E. Mann , and A. P. Williams . 2023. “Climate Change Will Accelerate the High‐End Risk of Compound Drought and Heatwave Events.” Proceedings of the National Academy of Sciences of the United States of America 120, no. 28: e2219825120. 10.1073/pnas.2219825120. 37399379 PMC10334742

[gcb71005-bib-0112] Tubiello, F. N. , J.‐F. Soussana , and S. M. Howden . 2007. “Crop and Pasture Response to Climate Change.” Proceedings of the National Academy of Sciences 104, no. 50: 19686–19690. 10.1073/pnas.0701728104. PMC2148358 18077401

[gcb71005-bib-0113] Unger, S. , C. Máguas , J. S. Pereira , T. S. David , and C. Werner . 2010. “The Influence of Precipitation Pulses on Soil Respiration—Assessing the ‘Birch Effect’ by Stable Carbon Isotopes.” Soil Biology and Biochemistry 42, no. 10: 1800–1810. 10.1016/j.soilbio.2010.06.019.

[gcb71005-bib-0114] Vargas, R. , P. E. Sánchez‐Cañete , P. Serrano‐Ortiz , et al. 2018. “Hot‐Moments of Soil CO_2_ Efflux in a Water‐Limited Grassland.” Soil Systems 2, no. 3: 47. 10.3390/soilsystems2030047.

[gcb71005-bib-0115] Vidal, A. , J. Hirte , S. F. Bender , et al. 2018. “Linking 3D Soil Structure and Plant‐Microbe‐Soil Carbon Transfer in the Rhizosphere.” Frontiers in Environmental Science 6: 9. 10.3389/fenvs.2018.00009.

[gcb71005-bib-0116] Walker, T. W. N. , C. Kaiser , F. Strasser , et al. 2018. “Microbial Temperature Sensitivity and Biomass Change Explain Soil Carbon Loss With Warming.” Nature Climate Change 8, no. 10: 885–889. 10.1038/s41558-018-0259-x. PMC6166784 30288176

[gcb71005-bib-0117] Wang, Y. , Y. Hao , X. Y. Cui , et al. 2014. “Responses of Soil Respiration and Its Components to Drought Stress.” Journal of Soils and Sediments 14, no. 1: 99–109. 10.1007/s11368-013-0799-7.

[gcb71005-bib-0118] Waring, B. G. , and J. S. Powers . 2016. “Unraveling the Mechanisms Underlying Pulse Dynamics of Soil Respiration in Tropical Dry Forests.” Environmental Research Letters 11, no. 10: 105005. 10.1088/1748-9326/11/10/105005.

[gcb71005-bib-0119] Warren, C. R. , and S. Manzoni . 2023. “When Dry Soil Is Re‐Wet, Trehalose Is Respired Instead of Supporting Microbial Growth.” Soil Biology and Biochemistry 184: 109121. 10.1016/j.soilbio.2023.109121.

[gcb71005-bib-0120] Wen, T. , G.‐H. Yu , W.‐D. Hong , et al. 2022. “Root Exudate Chemistry Affects Soil Carbon Mobilization via Microbial Community Reassembly.” Fundamental Research 2, no. 5: 697–707. 10.1016/j.fmre.2021.12.016. 38933120 PMC11197519

[gcb71005-bib-0121] Werth, M. , and Y. Kuzyakov . 2008. “Root‐Derived Carbon in Soil Respiration and Microbial Biomass Determined by 14C and 13C.” Soil Biology and Biochemistry 40, no. 3: 625–637. 10.1016/j.soilbio.2007.09.022.

[gcb71005-bib-0122] Widanagamage, N. , E. Santos , C. W. Rice , and A. Patrignani . 2025. “Study of Soil Heterotrophic Respiration as a Function of Soil Moisture Under Different Land Covers.” Soil Biology and Biochemistry 200: 109593. 10.1016/j.soilbio.2024.109593.

[gcb71005-bib-0123] Xiang, S.‐R. , A. Doyle , P. A. Holden , and J. P. Schimel . 2008. “Drying and Rewetting Effects on C and N Mineralization and Microbial Activity in Surface and Subsurface California Grassland Soils.” Soil Biology & Biochemistry, Special Section: Enzymes in the Environment 40, no. 9: 2281–2289. 10.1016/j.soilbio.2008.05.004.

[gcb71005-bib-0124] Yang, J. , X. Jia , H. Ma , et al. 2022. “Effects of Warming and Precipitation Changes on Soil GHG Fluxes: A Meta‐Analysis.” Science of the Total Environment 827: 154351. 10.1016/j.scitotenv.2022.154351. 35259374

[gcb71005-bib-0125] Yang, L. , J. Pan , J. Wang , et al. 2023. “Soil Microbial Respiration Adapts to Higher and Longer Warming Experiments at the Global Scale.” Environmental Research Letters 18, no. 3: 034044. 10.1088/1748-9326/acbecb.

[gcb71005-bib-0126] Yu, H. , X. Liu , Q. Ma , et al. 2021. “Climatic Warming Enhances Soil Respiration Resilience in an Arid Ecosystem.” Science of the Total Environment 756: 144005. 10.1016/j.scitotenv.2020.144005. 33277014

[gcb71005-bib-0127] Yuste, J. C. , I. A. Janssens , A. Carrara , L. Meiresonne , and R. Ceulemans . 2003. “Interactive Effects of Temperature and Precipitation on Soil Respiration in a Temperate Maritime Pine Forest.” Tree Physiology 23, no. 18: 1263–1270. 10.1093/treephys/23.18.1263. 14652226

[gcb71005-bib-0128] Zhang, G. , G. Zhou , X. Zhou , et al. 2023. “Effects of Tree Mycorrhizal Type on Soil Respiration and Carbon Stock via Fine Root Biomass and Litter Dynamic in Tropical Plantations.” Journal of Plant Ecology 16, no. 1: rtac056. 10.1093/jpe/rtac056.

[gcb71005-bib-0129] Zhang, S. , M. Wang , J. Zheng , and Z. Luo . 2026. “A Global Dataset of Soil Organic Carbon Mineralization in Response to Incubation Temperature Changes.” Earth System Science Data 18, no. 1: 131–146. 10.5194/essd-18-131-2026.

[gcb71005-bib-0130] Zhang, X. , N. Bilyera , L. Fan , et al. 2023. “The Spatial Distribution of Rhizosphere Microbial Activities Under Drought: Water Availability Is More Important Than Root‐Hair‐Controlled Exudation.” New Phytologist 237, no. 3: 780–792. 10.1111/nph.18409. 35986650

[gcb71005-bib-0131] Zhang, Z. , Y. Li , R. A. Williams , et al. 2023. “Responses of Soil Respiration and Its Sensitivities to Temperature and Precipitation: A Meta‐Analysis.” Ecological Informatics 75: 102057. 10.1016/j.ecoinf.2023.102057.

[gcb71005-bib-0132] Zhang, Z. , D. Wang , and M. Li . 2022. “Soil Respiration, Aggregate Stability and Nutrient Availability Affected by Drying Duration and Drying‐Rewetting Frequency.” Geoderma 413: 115743. 10.1016/j.geoderma.2022.115743.

[gcb71005-bib-0133] Zhao, J. , R. Li , X. Li , and L. Tian . 2017. “Environmental Controls on Soil Respiration in Alpine Meadow Along a Large Altitudinal Gradient on the Central Tibetan Plateau.” Catena 159: 84–92. 10.1016/j.catena.2017.08.007.

[gcb71005-bib-0134] Zhou, W. , D. Hui , and W. Shen . 2014. “Effects of Soil Moisture on the Temperature Sensitivity of Soil Heterotrophic Respiration: A Laboratory Incubation Study.” PLoS One 9, no. 3: e92531. 10.1371/journal.pone.0092531. 24647610 PMC3960259

