# Supplementary material for: Drought and Warming‐Induced Drying Suppress Soil Respiration but Amplify Rewetting‐Induced Pulses in a Temperate Pasture
Source: Glob Chang Biol. 2026 Jul 18;32(7):e71005. doi: 10.1111/gcb.71005 (PMC13380323; doi:10.1111/gcb.71005)
Supplement: Supplementary file 1 — Figure S1: Experimental shelter at the PAstures and Climate Extremes (PACE) facility (Richmond, New South Wales, Australia, −33.60972, 150.73833, 25 m asl), illustrating the infrastructure used for field‐based climate manipulation. Figure S2: Simulated rainfall in Dry and Wet treatment across the study period. Figure S3: (A) Soil respiration‐temperature and (B) soil respiration‐moisture relationships. Figure S4: Effects of drought and warming on aboveground and belowground biomass. Figure S5: Effects of drought and warming on soil physicochemical parameters in non‐rhizosphere (hatched) and rhizosphere (dotted) in 0–5 cm and 5–10 cm depth. Figure S6: Effects of drought and warming on microbial extracellular enzyme activity in non‐rhizosphere and rhizosphere in 0–5 cm and 5–10 cm depth. Figure S7: Relationship between soil respiration (SR) and aboveground biomass (AGB) across different treatments. Figure S8: Soil water content (SWC) response to rewetting. Table S1: Seasonal rainfall and total rainfall events in Wet and Dry treatments during 2024. Table S2: Drought and Warming effects on SRoverall, soil microclimate, aboveground biomass and rewetting response parameters. [file GCB-32-e71005-s001.docx]

**Drought and warming-induced drying suppress soil respiration but amplify rewetting-induced pulses in a temperate pasture**

**Running Title: Soil respiration under climate extremes**

Pankaj Tiwari^1*^, Elise Pendall^1*^, Nicholas Wright-Osment^1,2^, Nor Azizah Kusai^1^, Manjunatha H. Chandregowda^1^, Awais Shakoor^1^, Debjani Sihi^3^, Sally A. Power^1^, Eric A. Davidson^4^, Catriona A. Macdonald^1,5^

**Institutional affiliation:**

^1^Hawkesbury Institute for the Environment, Western Sydney University, Locked Bag 1797, Penrith, NSW 2751, Australia

^2^Department of Environmental Systems Science, ETH Zürich, 8092 Zürich, Switzerland

^3^Department of Plant and Microbial Biology and Crop and Soil Sciences, N.C. Plant Sciences Initiative, North Carolina State University, Raleigh, NC, USA

^4^Appalachian Laboratory, University of Maryland Center for Environmental Science, Frostburg, MD, USA

^5^School of Agriculture and Environment, University of Western Australia, Perth, WA 6090, Australia

***Authors for correspondence:**

1. Elise Pendall: [e.pendall@westernsydney.edu.au](mailto:e.pendall@westernsydney.edu.au),

2. Pankaj Tiwari: [p.tiwari@westernsydney.edu.au](mailto:p.tiwari@westernsydney.edu.au), [panktiwari8@gmail.com](mailto:panktiwari8@gmail.com)

**
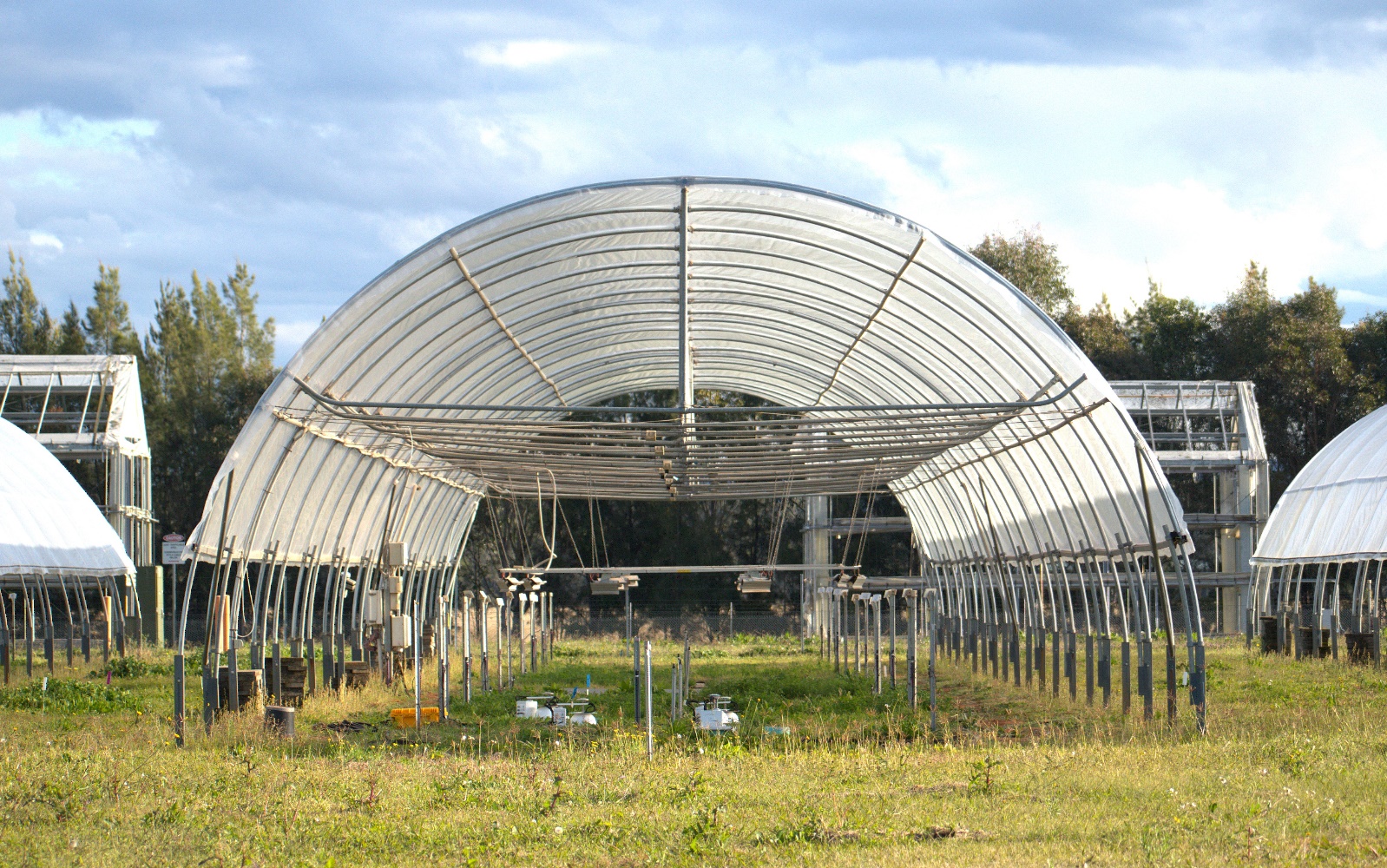
**

**Figure S1.** Experimental shelter at the PAstures and Climate Extremes (PACE) facility (Richmond, New South Wales, Australia, -33.60972, 150.73833, 25 m asl), illustrating the infrastructure used for field-based climate manipulation.

**
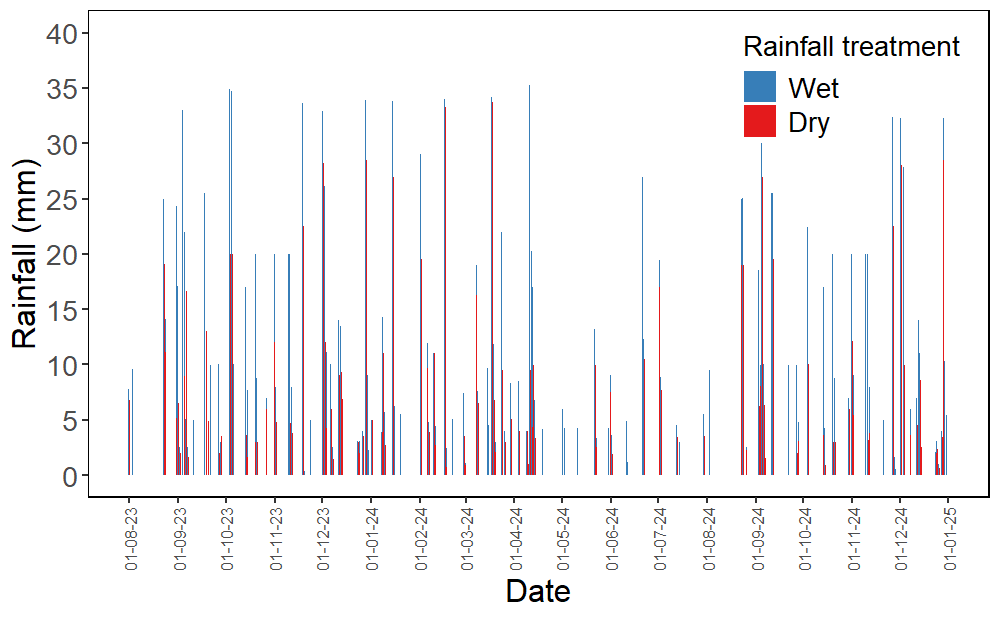
**

**Figure S2. Simulated rainfall in Dry and Wet treatment across the study period.** Dry plots received 52%, 29%, 50%, and 33%, less rainfall than Wet plots in spring, summer, autumn, and winter, respectively, resulting in an overall annual (year 2024) difference in rainfall of 42% between Wet and Dry regimes treatment (Figure S2, Table S1).


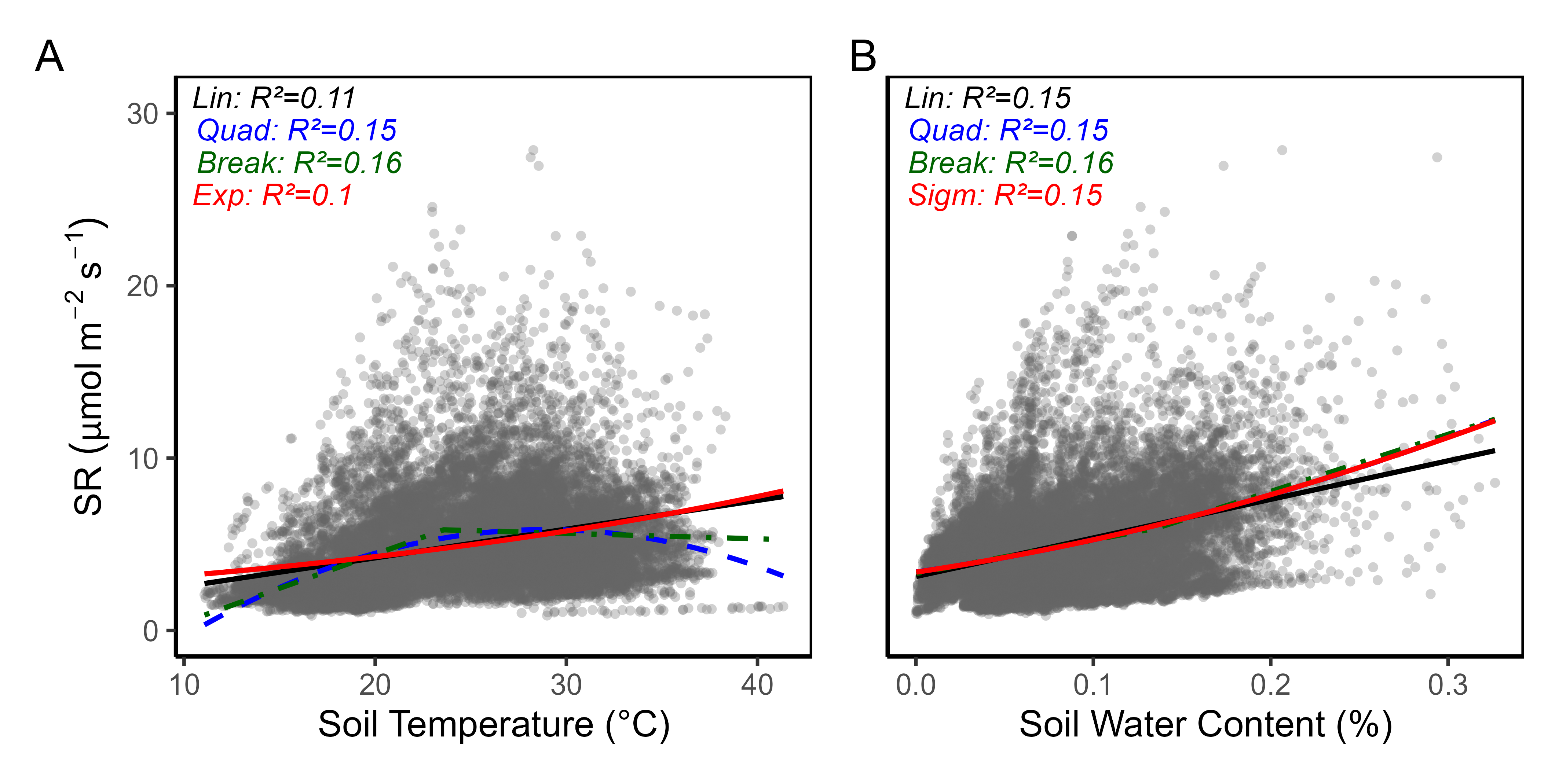


**Figure S3. (A) Soil respiration-temperature and (B) soil respiration-moisture relationships.** Relationships were evaluated using linear, quadratic, broken-stick, exponential, and sigmoidal models. Points represent observed data, and lines represent model predictions.


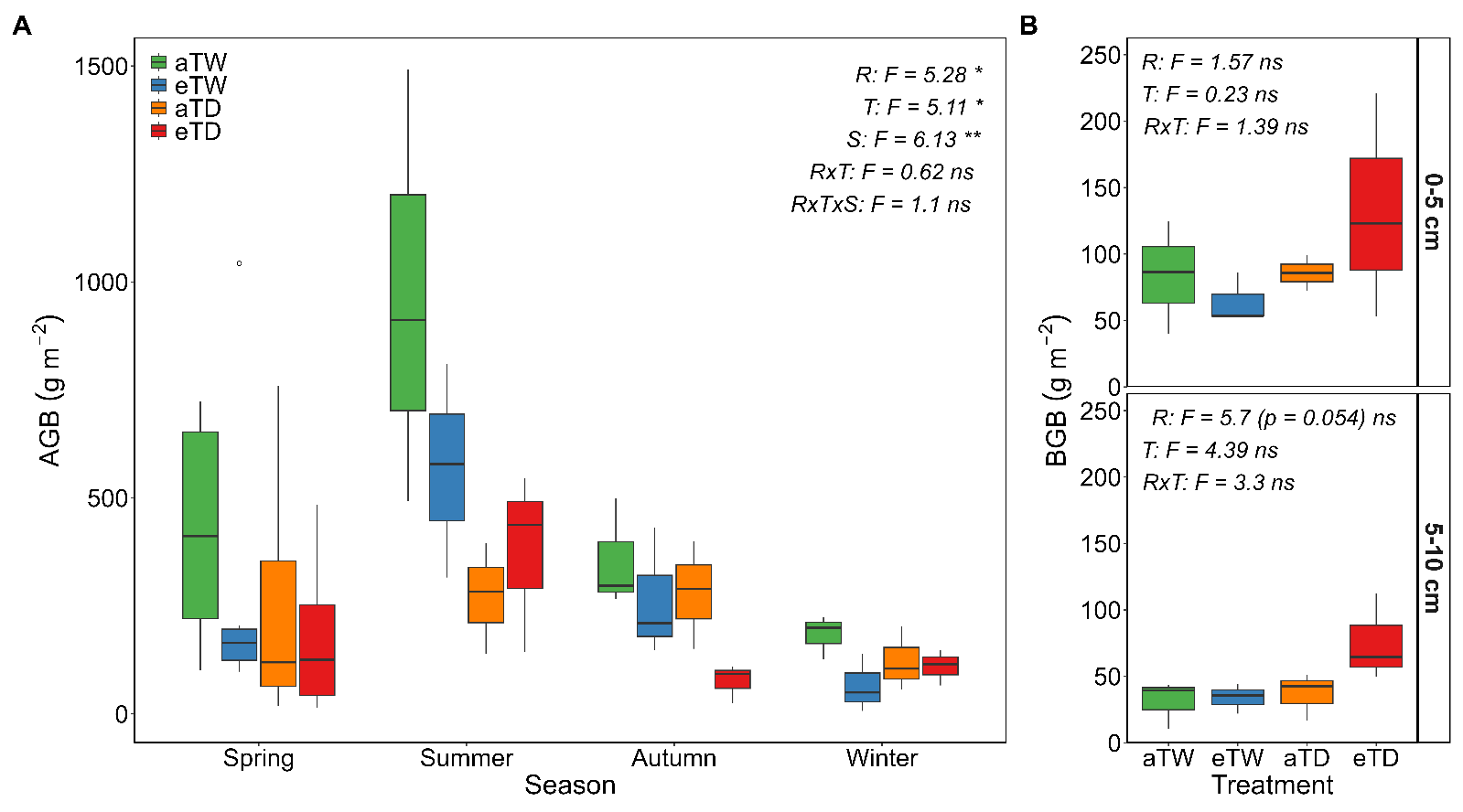


**Figure S4. Effects of drought and warming on aboveground** **and belowground biomass. (A)** Total aboveground biomass clipped from inside soil collars before SR measurements across different seasons. **(B**) Fine root biomass at 0-5 cm and 5-10 cm. Horizontal lines within boxes indicate medians, and the upper and lower edges of the box plots represent the 25th and 75th percentiles. The top bar shows the maximum value, and the bottom bar the minimum value. The effects were analysed based on linear mixed-effects models with F-values reported as insets. R, T and S represent rainfall, temperature and seasons, respectively. * *p* < 0.05, ***p* < 0.01, *** *p* < 0.001 and ns = non-significant. aTD, aTW, eTD & eTW, are treatments where, aT and eT stands for ambient and elevated temperature, and D and W represent Dry and Wet, respectively.


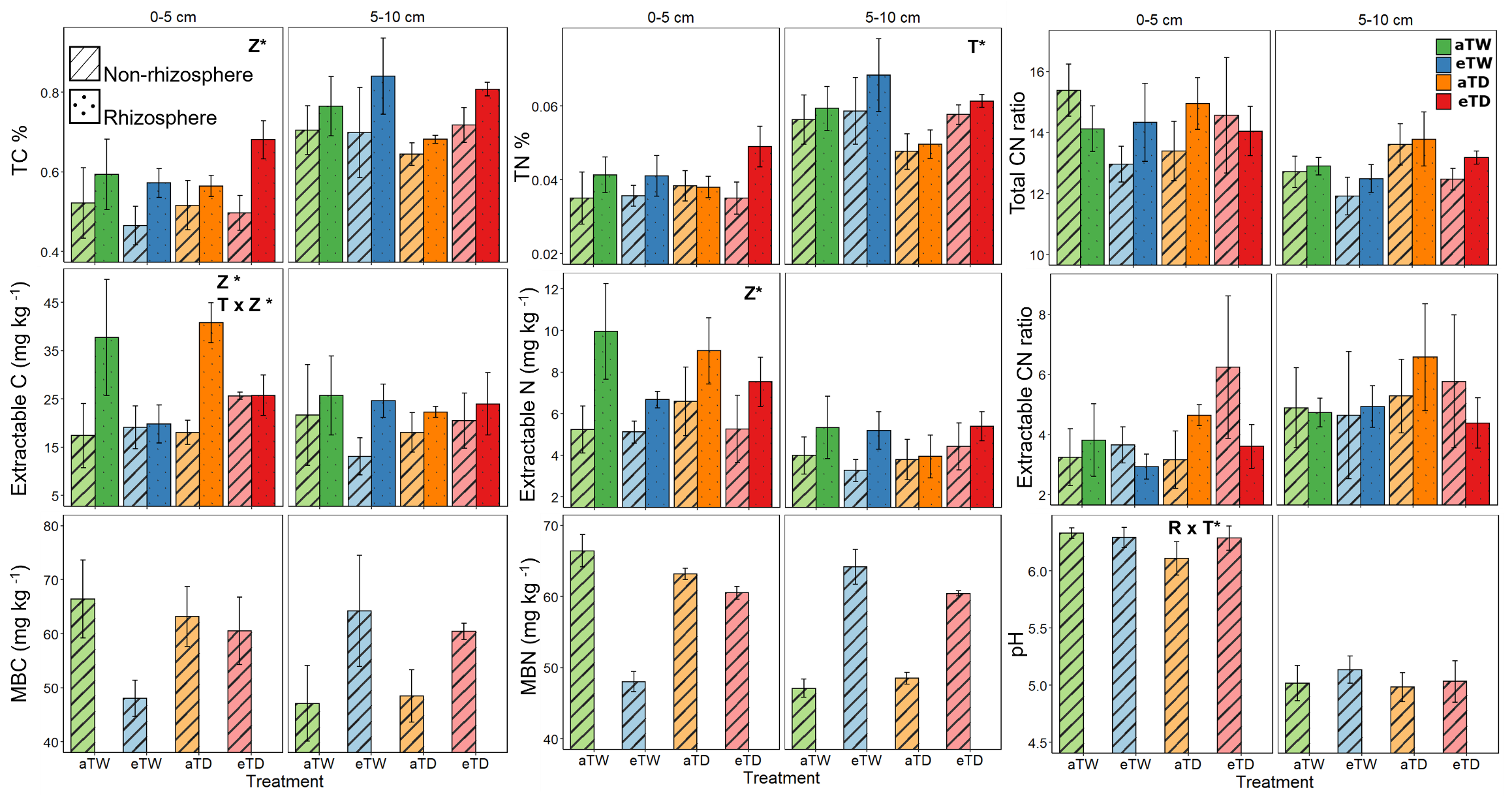


**Figure S5. Effects of drought and warming on soil physicochemical parameters in non-rhizosphere (hatched) and rhizosphere (dotted) in 0-5 cm and 5-10 cm depth.** MBC, MBN and pH were only assessed from non-rhizosphere soil. R, T and Z represent rainfall, temperature and zone (rhizosphere or non-rhizosphere), respectively. aTD, aTW, eTD & eTW are treatments, where, aT and eT stands for ambient and elevated temperature, and D and W represent Dry and Wet, respectively. Data shows mean ± standard error of mean during March 2024. * *p* < 0.05, ***p* < 0.01, *** *p* < 0.001 and ns = non-significant. The effects of Rainfall, Temperature and Zone were analysed across each depth based on linear mixed-effects models.


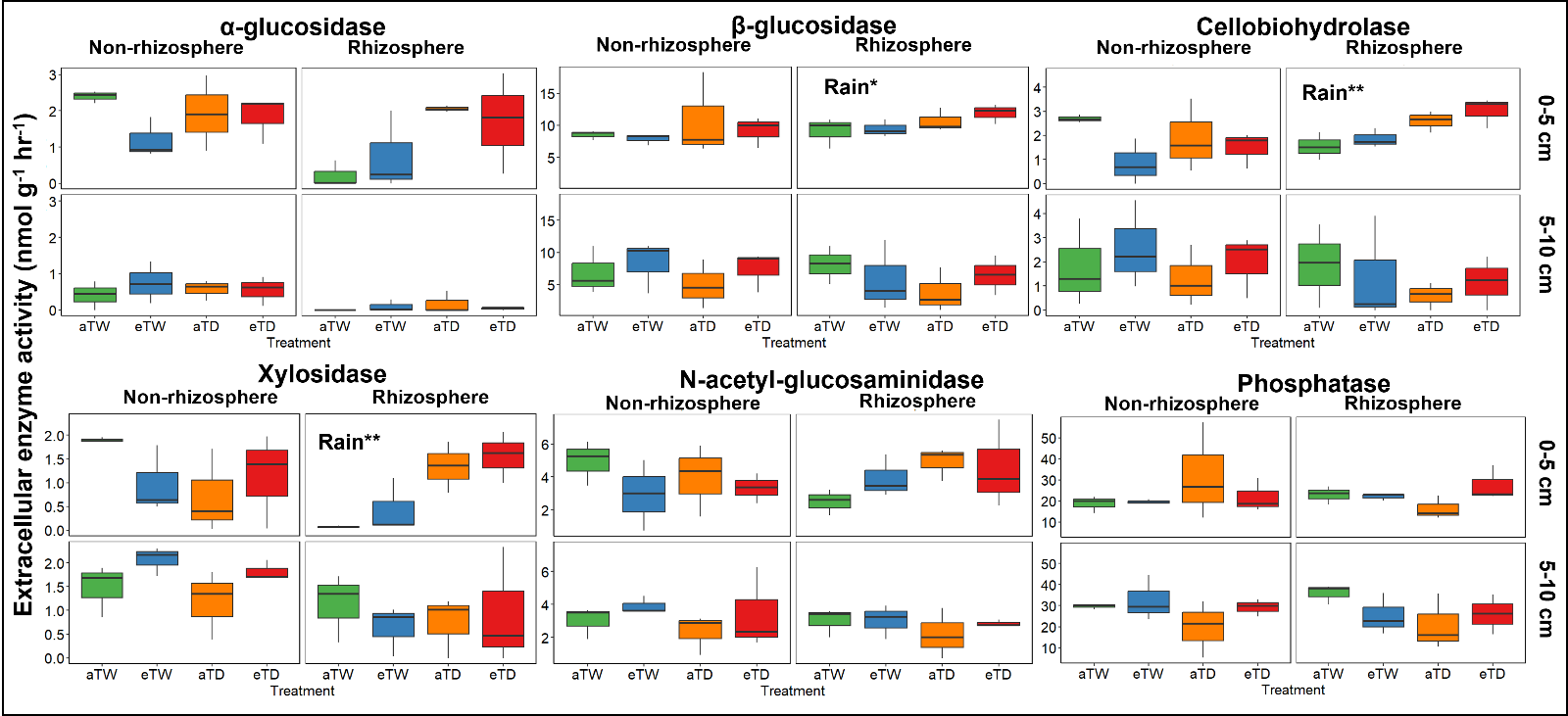


**Figure S6. Effects of drought and warming on microbial extracellular enzyme activity in non-rhizosphere and rhizosphere in 0-5 cm and 5-10 cm depth.** Effects of Rainfall and Temperature were tested using linear mixed-effects models across each zone and depth separately. * *p* < 0.05, ***p* < 0.01, *** *p* < 0.001 and ns = non-significant. Horizontal lines within boxes indicate medians, and the upper and lower edges of the box plots represent the 25th and 75th percentiles. The top bar shows the maximum value, and the bottom bar the minimum value. aTD, aTW, eTD & eTW, are treatments where, aT and eT stands for ambient and elevated temperature, and D and W represent Dry and Wet, respectively.


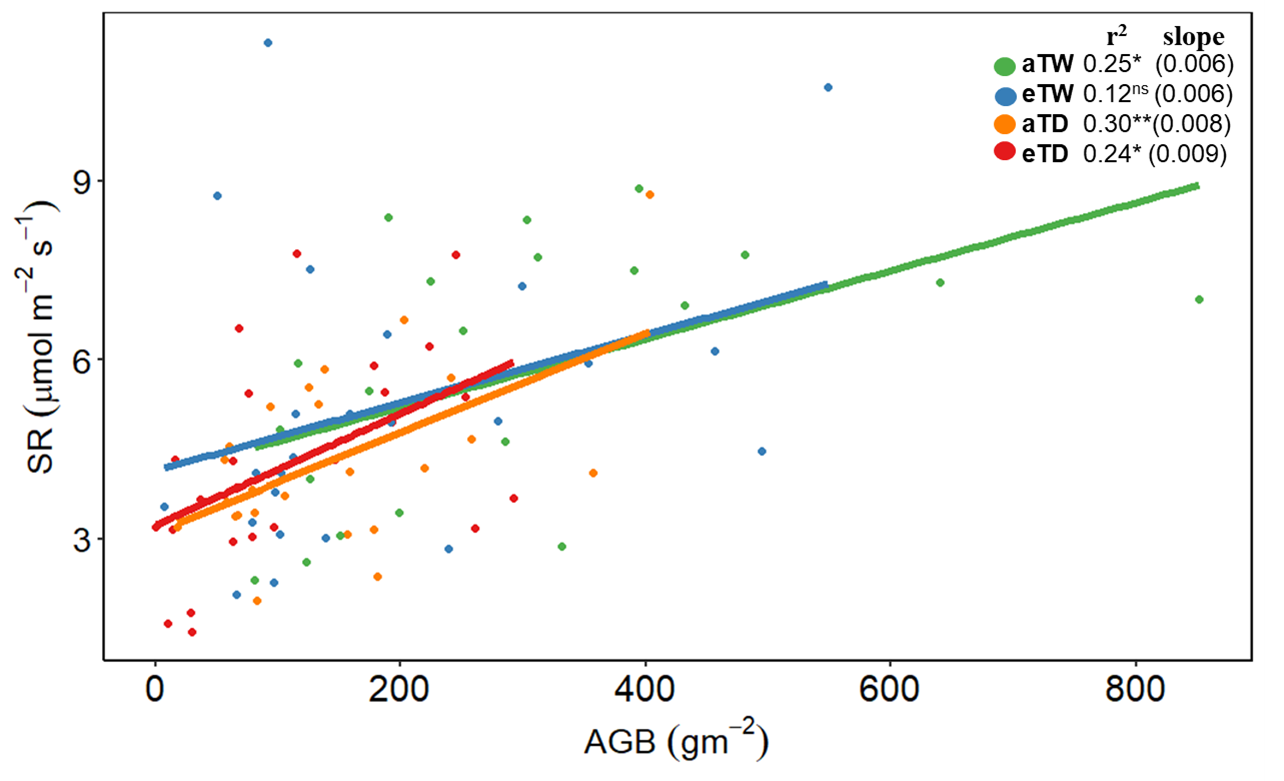


**Figure S7. Relationship between soil respiration (SR) and aboveground biomass (AGB) across different treatments.** Linear relationship between mean SR at each campaign and aboveground biomass clipped from inside soil collars prior to SR measurements. *** *p<*0.001, ** p<0.01, * p<0.05 and ns represent non-significant. aTD, aTW, eTD & eTW are treatments where, aT and eT stands for ambient and elevated temperature, and D and W represent Dry and Wet, respectively.


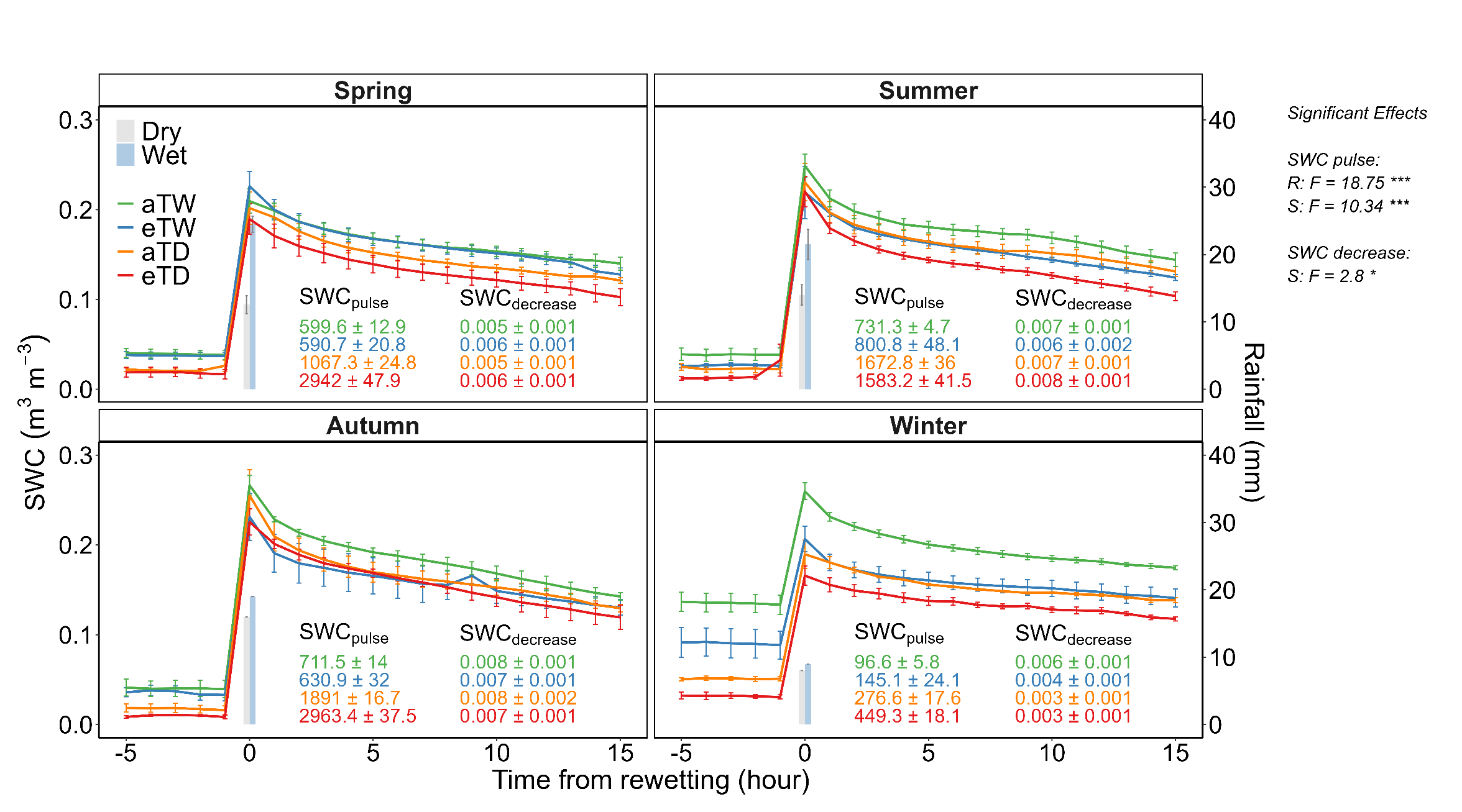


**Figure S8. Soil water content (SWC) response to rewetting.** SWC_pulse_ represents increase in SWC (%) upon rewetting while SWC_decrease_ is the rate of decrease in SWC (m⁻^3^ m⁻^3^ hr⁻^1^) after peak. Colours depict the respective treatment aTD, aTW, eTD & eTW, where, aT and eT stands for ambient and elevated temperature, and D and W represent Dry and Wet, respectively. Each data point shows mean SWC across campaigns and soil collars for each season; bars show standard errors. The effects were analysed based on linear mixed-effects models with F-values reported as insets. R and S represent rainfall and seasons, respectively. No significant temperature effect was observed. * *p* < 0.05 and *** *p* < 0.001.

**Table S1. Seasonal rainfall and total rainfall events in Wet and Dry treatments during 2024.**

|  |  |  |  |
| --- | --- | --- | --- |
| **Season** | **Treatment** | **Events** | **Total rain (mm)** |
| Spring | Wet | 20 | 315.7 |
| Summer | Wet | 24 | 325.1 |
| Autumn | Wet | 20 | 244.0 |
| Winter | Wet | 10 | 112.9 |
| Spring | Dry | 15 | 151.0 |
| Summer | Dry | 20 | 229.4 |
| Autumn | Dry | 11 | 121.8 |
| Winter | Dry | 7 | 76.0 |

**Table S2. Drought and Warming effects on SR_overall_, soil microclimate, aboveground biomass and rewetting response parameters.** Abbreviations: SR, soil respiration; SWC, soil water content; ST, soil temperature; AGB, aboveground biomass; SR_pulse_, SR increase upon rewetting; Norm. SR_pulse_, rain-normalized SR_pulse_; SR_peak_, peak SR upon rewetting; SR_decrease_, SR decrease rate post pulse. Values represent estimated marginal means obtained from post-hoc pairwise comparisons of linear mixed-effects models. Significance levels: *p* < 0.05, *p* < 0.01, *p* < 0.001; ns, not significant. Data transformations applied prior to analysis are indicated in brackets alongside the corresponding parameters. Pairwise comparisons were conducted only for parameters where significant interactions were detected in Table 1. aT and eT stands for ambient and elevated temperature, and D and W represent Dry and Wet, respectively.

|  |  |  |  |  |  |  |
| --- | --- | --- | --- | --- | --- | --- |
| **Factor** | **Season** | **Drought effect** | | **Warming effect** | | **Interaction** |
|  |  | aT | eT | Wet | Dry | **Contrast** |
| **SR_overall_ (log)** | Spring | -0.209 ± 0.012 *** | -0.191 ± 0.012 *** | -0.087 ± 0.012 *** | -0.105 ± 0.012 *** | -0.018 ± 0.017 ns |
|  | Summer | -0.539 ± 0.015 *** | -0.189 ± 0.015 *** | 0.145 ± 0.015 *** | -0.204 ± 0.015 *** | -0.350 ± 0.021 *** |
|  | Autumn | -0.226 ± 0.019 *** | -0.282 ± 0.019 *** | -0.249 ± 0.019 *** | -0.193 ± 0.019 *** | 0.056 ± 0.026 * |
|  | Winter | -0.089 ± 0.019 *** | -0.304 ± 0.019 *** | -0.323 ± 0.019 *** | -0.108 ± 0.019 *** | 0.215 ± 0.027 *** |
| **SWC (sqrt)** | Spring | -0.038 ± 0.002 *** | -0.044 ± 0.002 *** | -0.022 ± 0.002 *** | -0.016 ± 0.002 *** | 0.006 ± 0.003 ns |
|  | Summer | -0.062 ± 0.003 *** | -0.050 ± 0.003 *** | -0.034 ± 0.003 *** | -0.047 ± 0.003 *** | -0.013 ± 0.004 ** |
|  | Autumn | -0.043 ± 0.004 *** | -0.050 ± 0.004 *** | -0.030 ± 0.004 *** | -0.024 ± 0.004 *** | 0.007 ± 0.005 ns |
|  | Winter | -0.065 ± 0.004 *** | -0.050 ± 0.004 *** | -0.026 ± 0.004 *** | -0.041 ± 0.004 *** | -0.015 ± 0.005 ** |
| **ST (raw)** | Overall | 0.372 ± 0.109 *** | 0.836 ± 0.109 *** | 3.042 ± 0.109 *** | 2.577 ± 0.109 *** | -0.464 ± 0.154 ** |
| **AGB (log)** | Overall | -0.562 ± 0.244 ** | | -0.552 ± 0.244 * | | ns |
| **SR_pulse_ (sqrt)** | Overall | 2.259 ± 0.777 ** | | 3.783 ± 0.777 *** | | ns |
| **Norm. SR_pulse_ (raw)** | Spring | 13.914 ± 1.639 *** | | 7.031 ± 1.344 *** | | ns |
|  | Summer | 6.213 ± 2.291 ** | |  |  | ns |
|  | Autumn | 5.555 ± 3.240 ns | |  |  | ns |
|  | Winter | 9.183 ± 3.240 ** | |  |  | ns |
| **SR_peak_ (log)** | Overall | -0.105 ± 0.086 ns | | 0.158 ± 0.086 ns | | ns |
| **SR_decrease_ (raw)** | Overall | -0.021 ± 0.051 ns | | 0.139 ± 0.051 ** | | ns |
